# Towards an efficient validation of dynamical whole-brain models

**DOI:** 10.1038/s41598-022-07860-7

**Published:** 2022-03-14

**Authors:** Kevin J. Wischnewski, Simon B. Eickhoff, Viktor K. Jirsa, Oleksandr V. Popovych

**Affiliations:** 1 Institute of Neuroscience and Medicine – Brain and Behaviour (INM-7), Forschungszentrum Jülich, Jülich, Germany; 2 Institute of Systems Neuroscience, Heinrich Heine University Düsseldorf, Düsseldorf, Germany; 3 Institut de Neurosciences des Systèmes (INS, UMR1106), Inserm, Aix-Marseille University, Marseille, France

**Keywords:** Computational neuroscience, Mathematics and computing

## Abstract

Simulating the resting-state brain dynamics via mathematical whole-brain models requires an optimal selection of parameters, which determine the model’s capability to replicate empirical data. Since the parameter optimization via a grid search (GS) becomes unfeasible for high-dimensional models, we evaluate several alternative approaches to maximize the correspondence between simulated and empirical functional connectivity. A dense GS serves as a benchmark to assess the performance of four optimization schemes: Nelder-Mead Algorithm (NMA), Particle Swarm Optimization (PSO), Covariance Matrix Adaptation Evolution Strategy (CMAES) and Bayesian Optimization (BO). To compare them, we employ an ensemble of coupled phase oscillators built upon individual empirical structural connectivity of 105 healthy subjects. We determine optimal model parameters from two- and three-dimensional parameter spaces and show that the overall fitting quality of the tested methods can compete with the GS. There are, however, marked differences in the required computational resources and stability properties, which we also investigate before proposing CMAES and BO as efficient alternatives to a high-dimensional GS. For the three-dimensional case, these methods generated similar results as the GS, but within less than 6% of the computation time. Our results contribute to an efficient validation of models for personalized simulations of brain dynamics.

## Introduction

Following the ground-breaking work of Biswal et al.^1^, neuroimaging research has experienced a shift in attention towards resting-state brain activity^2,3^. The discovered similarity between the task-evoked functional networks and the corresponding connectivity patterns observed from human brain activity at rest strongly motivated the investigation of the latter^1,4,5^. A multitude of study approaches and applications of the resting-state dynamics have been developed. They aimed at understanding the brain architecture and function on the one hand and at a differentiation between individuals in health and disease on the other hand^6–12^. A promising avenue to achieve both of these goals is provided by numerical simulations of complex spatio-temporal brain activity patterns via dynamical whole-brain models^13–19^.

The data-driven dynamical models allow for the incorporation of anatomical information about the human brain into the simulation of its dynamical properties. In other words, they enable researchers to investigate the relationship between brain structure and function with a particular focus on the question whether and how the latter emerges from the former and how they correlate^13–19^. Additionally, models offer a quick in silico experimental way to study and compare different brain parcellations, network configurations and parameters of data-preprocessing, which again contributes to a deeper understanding of the interaction between the brain architecture and dynamics^20–22^. Another advantage of the discussed modeling approach is that it allows for a meaningful description of complex natural phenomena by a set of interpretable parameters. The whole-brain models are designed to mimic human brain dynamics in a way that fairly reduces its intricacy and renders it accessible for deep and systematic analyses^23^. Differentiation between individuals in health and disease can thus be facilitated on the basis of biologically motivated model parameters and model dynamics. Model simulations can also improve the overall interpretability by performing a certain denoising, meaning that only the relevant dynamics and phenomena are modeled, but not the unwanted effects of random noise. In particular, model simulations showed a potential to reveal previously unknown characteristics of neural activity, allowing, for example, to distinguish between disease subtypes which cannot be identified by considering empirical brain imaging data only^24^.

Current understanding of the mechanisms underlying the observed complex patterns of collective neuronal dynamics and connectivity remains, however, highly fragmented, and deeper investigations are still required. In this effort, it is crucial to seize the full potential of the broad spectrum of available computational models^25–31^ developed in the course of brain research. Notably, with a rising number of neural activity models on several scales (single neurons, neuronal populations, brain regions) and their complexity, the challenge of their adequate validation against empirical data has become apparent. More precisely, model validation consists of adjusting the parameters, which have a well-founded relation to brain characteristics, e.g. global and local coupling, signal transmission delay or an excitation-inhibition balance^14,15,19^, in a way that the best possible correspondence between simulated and empirical dynamics is obtained. It is of highest relevance for recent investigations of personalized brain modeling, where a search for subject-specific optimal parameters is conducted^32–35^. Additionally, the application of individualized brain models in population-based approaches offers a promising way to examine between-subject effects and possible parametrizations of inter-individual differences^24,36^.

An intuitive approach is to optimize model parameters by a systematic exploration along a dense discrete grid in the parameter space. This procedure, often termed *grid search*, has been applied in a variety of model-based studies^16,35,37,38^. On first sight, such a brute force approach may appear to be the *safe* way to explore the parameter space, but it holds several conceptual and technical challenges. Foremost, the number of parameters used in a grid search is typically small (such as 2–5), where in the real system adjustments can be made for many more parameters. Arguments are evoked that these parameters can be estimated from data, which usually assumes independent measurements. As joint probabilities of parameters are not necessarily limited to second orders (see, for instance, the work of Marder and Goaillard^39^), degeneracy will be a major contaminating factor for a proper interpretation of the simulation results. The second conceptual challenge is the extraction of data features used for the comparison between simulated and empirical data. The selective use of simplifying data features facilitates the analysis, but also imposes theory-driven filters in interpreting the results. The technical challenges are computational. Given that the grid is dense enough, the probability to miss important characteristics (such as the maximal similarity between simulated and empirical data) is low. Unfortunately, an exploration of the entire parameter space on a dense grid is computationally expensive and becomes unfeasible for complex models with many free parameters. If, for example, a machine equipped with $p$ = 48 processors required only $t$ = 100 s per subject to perform a one-dimensional grid search covering $p$ parameter points, then the total time consumption of a grid search covering ${p}^{\text{Dim}}$ (= 110 592) parameter points for a model with ${\text{Dim}}$ = 3 free parameters would roughly be given as $t\,p^{{{\text{Dim}} - 1}}$ = 64 h. For a cohort of *S* = 100 subjects, the demands rise to $S\,t\,p^{{{\text{Dim}} - 1}}$, which corresponds to around 9 months of calculations. Another aspect, whose importance must not be neglected, is that the sheer mass of occurring parameter points renders the entire investigation intractable, too. That is why already a three-dimensional grid search with adequate granularity as, for example, in Schirner et al.^35^, is rather exceptional than usual. Additionally, the strategy of a sequential tuning of certain parameters^14,36^, where individual components are optimized separately from each other and afterwards fixed, may not always be applicable for multi-dimensional tasks that are not separable into low-dimensional subproblems. These aspects hamper high-dimensional model studies in their potential to infer parameter-dependent differences between subjects. Papers discussing individualized simulations of brain dynamics are therefore based on models that hardly ever exceed five free parameters, with a majority investigating up to three variables only. Studies bringing forward desirable enhancements to more than 100 region-specific model parameters, as described in Wang et al.^40^, for example, currently lack feasible ways of implementation.

Previous works^40–43^ have already identified the so-called *inverse problem* of estimating optimal parameters from given empirical data and advocated for the use of alternatives to a thorough parameter space scan. We emphasize that optimal parameters usually comprise degenerate manifolds that ideally would be sampled systematically by Monte Carlo approaches^32,33^, but the use of mathematical optimization algorithms is often the more practical solution^44–46^. These methods enable the exploration of high-dimensional parameter spaces (under certain limiting assumptions), for which a grid search is impracticable. In good practice, when aware of the limitations and underlying hypothesis (such as the existence of a unimodal posterior distribution), these approaches can be very powerful. Currently, the literature lacks an evaluation of the particular advantages and limitations connected to their applicability in individualized whole-brain modeling. Studies based on larger subject cohorts could underpin their robustness in dealing with in vivo data that feature increased complexity compared to purely theoretical and noise-free mathematical problems, which constitute a common field of their application.

In this work, we focus on the technical computational challenges. We implemented and tested several such optimization schemes which were designed to extensively explore complex high-dimensional parameter spaces in an adequate amount of computation time. Searching for subject-specific optima, we investigated the applicability of these techniques for the validation of whole-brain models. We systematically compared their outcomes as well as computational costs with each other and the grid search approach. In particular, we considered a system of phase oscillators with delayed coupling^47–49^ to model the resting-state blood oxygen level-dependent (BOLD) dynamics, from which we extracted the simulated data feature of functional connectivity (FC). For a cohort of 105 subjects, the model was built upon the individual anatomical brain connectivity obtained from diffusion-weighted magnetic resonance imaging (dwMRI). It determined the coupling weights and time delays between individual network nodes. The individual model was then validated against the corresponding functional neuroimaging data (empirical FC) for each subject. Model parameters were optimized in order to maximize the model fit given by the correlation between simulated and empirical FCs. The highest possible similarity between these quantities was termed the model’s *goodness-of-fit*.

We studied four derivative-free methods as given by the Nelder-Mead Algorithm^50^, Particle Swarm Optimization^51^, Covariance Matrix Adaptation Evolution Strategy^52,53^ and Bayesian Optimization^54^. These methods were directly applied to the simulation output obtained from the numerical integration of the model equations. Our investigations were made for two and three free parameters in the utilized model. To better assess the quality of the algorithm solutions, we also included a systematic parameter variation (grid search) for each subject in both dimensionalities of the parameter space. It served as a good approximation of a *ground truth* provided that the grid is dense enough in the considered range of parameters. The comparison criteria considered the obtained goodness-of-fit, the required computation time and the location of optimized parameters, or, more precisely, the spread of the algorithm solutions combined with their distance to the grid search optima.

Our study aims at suggesting efficient and reliable algorithms for the optimization of the correspondence of functional connectivity within simulated and empirical data in whole-brain modeling. The intention is to point up the ways that have to be taken in higher dimensions, where the grid search approach becomes inapplicable due to insurmountable numbers of parameter constellations that would have to be evaluated. After analyzing the advantages and drawbacks of the tested methods, we eventually arrive at a recommendation for two particular algorithms that provide a favorable trade-off between the result robustness and invested computational resources.

## Methods

### Overview

In this study, we worked with neuroimaging data of *S* = 105 healthy, unrelated subjects (age 28.5 ± 3.4 years, 51 males) from the Human Connectome Project (HCP) S1200 public dataset release^55^. Approval for the study was given by the local ethics committee of the HCP WU-Minn and written, informed consent was obtained from all subjects. All methods were performed in accordance with the relevant guidelines and regulations. The given datasets had been utilized for the extraction of structural and resting-state functional connectivity (SC and FC, respectively) in related works^20–22^. We used the obtained empirical connectomes for the model derivation and validation. More precisely, Schaefer’s atlas^56^ with *N* = 100 cortical regions was employed as a brain parcellation for the calculation of an atlas-based SC. The latter served as a basis for the underlying network in the dynamical model and was used to determine the coupling weights and delays between individual network nodes (brain regions or parcels). The model then was deployed to simulate the resting-state brain dynamics and eventually generate simulated FC (simFC). This in turn was fitted to the empirical FC (empFC) by adjusting up to three model parameters simultaneously: global coupling and delay in a two-dimensional parameter space (see also Cabral et al.^57,58^) and additionally the noise intensity when a three-dimensional scenario was considered (see also Deco et al.^14^).

Besides an extensive parameter sweep exploration on a dense grid, referred to as a grid search, we implemented four mathematical optimization algorithms in order to determine the optimal parameter values which maximize the similarity between simulated and empirical data. We measured this similarity with the Pearson correlation coefficient between simFC and empFC. To compare the algorithms’ outcomes systematically with each other and the grid search, we focused on the following criteria: For every subject, we analyzed the goodness-of-fit values detected within a predefined number of algorithm executions with different initial conditions. The highest value as well as the standard deviation of all solutions were considered and compared across the methods. Then, the required computation time of the optimization algorithms was put in relation to the grid search’s demands. In addition, we investigated the reliability of the results by examining the location of the obtained solutions in the parameter space. The spread of the supposed optima detected by a given algorithm for a particular subject was analyzed together with the averaged distance to the respective optimal parameters found by the grid search. We want to highlight that, despite the important aspect of a reduced computation time, we were also concerned with finding stable methods that detect the global optima most reliably (which may be manifested by a low spread of solutions in the parameter space, for example). Our main goal was to suggest alternative ways for model validation that promise a clearly lower computational burden than the grid search combined with a favorable robustness. We thus analyzed the obtained results based on the three mentioned criteria, which can be summarized as

- the goodness-of-fit,
- the necessary computation time and
- the location/spread of the optimized parameters.

### Data preparation

The pipeline implemented for the extraction of SC comprised four major steps: preprocessing of raw magnetic resonance images (MRIs), calculation of a whole-brain tractography (WBT), transformation of the brain parcellation images to the native space and, finally, SC reconstruction based on the brain parcellation. In this procedure, software tools from ANTs^59^, FreeSurfer^60^, FSL^61^ and MRtrix3^62^ were applied, see Jung et al.^21^ and Popovych et al.^22^ for the details.

In the first step, the T1-weighted images and the diffusion-weighted MRIs (dwMRIs) were preprocessed (intensity normalization, denoising, motion correction, tissue segmentation, etc.) with functions from FreeSurfer and MRtrix3. Subsequently, a co-registration via FSL was performed. In the second step, functions from MRtrix3 were used to compute a WBT. A set of 10 M streamlines in total was extracted by the help of a probabilistic algorithm^63^. Afterwards, linear and non-linear transformations were applied to the image of Schaefer’s atlas in order to transfer it from the Montreal Neurological Institute (MNI) standard space, in which it was sampled, to the native diffusion space. The workflow in this step involved functions from FSL. Finally, the MRtrix3 function *tck2connectome* was used to reconstruct the parcellation-based SC that comprised two matrices: one with the number of streamlines between individual regions, i.e. the streamline count, henceforth referred to as SC, and another one containing the average length of the streamlines between regions, termed path lengths (PL).

For the calculation of empFC in turn, the ICA-FIX^64^ preprocessed resting-state functional MRI (fMRI) data provided by the HCP repository were considered. Time series of the BOLD signals were extracted as mean signals averaged over all voxels of individual brain regions. Then, calculating the Pearson correlation for each pair of linearly detrended and z-scored BOLD time series from the respective cortical areas eventually yielded the corresponding FC matrix.

In this setting, four fMRI sessions of 1200 volumes sampled with a repetition time of TR = 0.72 s each were available for every subject (scans were performed twice using different phase-encoding directions on two days). Additionally, a concatenated BOLD signal was generated by combining the z-scored BOLD time series from the mentioned sessions. We focused on the empFC matrix derived from the concatenated session for the optimization of model parameters in terms of the best fit between simulated and empirical data.

### Model simulations

To obtain simFC, we implemented the Kuramoto model of coupled phase oscillators^48,49,57^, which is one of the simplest models used for simulating features of resting-state brain activity (here the phase dynamics)^37^. Its reduced complexity compared to other whole-brain models makes it suitable to investigate the immediate impact of the model parameters on the simulated dynamics. We do not suspect, however, that our insights and qualitative results depend on the selected modeling approach. We therefore worked with this basic model, in which each of *N* oscillators represented one brain region defined by the underlying parcellation (*N* = 100 for the considered atlas). Their temporal dynamics served as a basis to compute simulated BOLD signals that were eventually cross-correlated in order to derive the simFC matrix. More precisely, the model assumes that the phase dynamics of the mean BOLD signal of a brain region *i* $\in$ {1, …, *N*} can be described by the following differential equation:

1 $$\dot{\theta }_{i} \left( t \right) = 2\pi f_{i} + \mathop \sum \limits_{j = 1}^{N} k_{ij} \sin \left( {\theta_{j} \left( {t - \tau_{ij} } \right) - \theta_{i} \left( t \right)} \right) + \sigma \eta_{i} \left( t \right).$$

For a given time *t*, *θ*_*i*_(*t*) $\in$ [0, 2*π*] denotes the value of the phase that oscillates with a natural frequency *f*_*i*_ if left uncoupled (*k*_*ij*_ = 0) from the other oscillators. The frequencies *f*_*i*_ were estimated from the maximal spectral peaks (restricted to the frequency range from 0.01 Hz to 0.1 Hz) of the empirical BOLD time series.

Apart from intrinsic oscillations, the phase dynamics is governed by delayed interactions with the other oscillators. The individual coupling strengths *k*_*ij*_ and delay values *τ*_*ij*_ were determined on the basis of the empirical matrices ${\text{SC}} = \left( {{\text{SC}}} \right)_{1 \le i,j \le N}$ and ${\text{PL}} = \left( {{\text{PL}}} \right)_{1 \le i,j \le N}$, respectively:

2 $$k_{ij} = \frac{{{\text{SC}}_{ij} }}{{{\langle\text{SC}\rangle}}}\frac{C}{N},\quad \tau_{ij} = \frac{{{\text{PL}}_{ij} }}{{{\langle\text{PL}\rangle}}}\tau .$$

In this context, $\left\langle \cdot \right\rangle$ indicates the mean over all matrix elements, excluding the zero diagonals. *C* and *τ* are global weighting factors that influence the strength and delay of the interactions between individual oscillators, respectively. These quantities can be interpreted as free parameters in the dynamical system. Additionally, it is possible to adjust the intensity *σ* of the independent noise *η*_*i*_(*t*) which is sampled from a uniform distribution over [− 1, 1] and perturbs the individual oscillators. While *C* and *σ* can be regarded as dimensionless quantities, *τ* is measured in seconds.

We solved Eq. (1) numerically using Heun’s method for stochastic differential equations^65^ with a discrete time step of Δ*t* = 0.06 s. The calculated phases were downsampled to 0.72 s in order to match the repetition time of the fMRI data provided by the HCP. With the model at hand, we generated simulated BOLD time series by computing $\sin \left( {\theta_{i} \left( t \right)} \right)$ for all *i* $\in$ {1, …, *N*} corresponding to the regions in the considered brain parcellation. As mentioned above, the matrix simFC was then derived from the Pearson correlation coefficients across all pairs of simulated time series. These featured a length of 3500 s after excluding an initial transient of 500 s. We assessed the similarity between simulated and empirical data by correlating the matrices of simFC = simFC(*C*, *τ*, *σ*) and empFC.

### Parameter optimization

Mathematically, the search for the parameters that maximize the correspondence between simFC and empFC (the model fit) can be interpreted as an optimization problem of a goal function *F* = *F*(*C*, *τ*, *σ*) that returns the Pearson correlation coefficient between the model output and the corresponding empirical measurement. Our knowledge about *F* is limited to a numerical calculation of its values (numerical integration of Eq. (1) and correlation between simFC and empFC) depending on the relevant model parameters (e.g. global coupling *C*, global delay *τ* and noise intensity *σ*) and the assumption of continuity. We thus supposed that the function *F* is not available in a closed form, and no claim was made about the existence of derivatives. In other words, we were to a great extent dealing with a black-box optimization problem. We optimized the model parameters by a systematic variation on a dense grid, before testing the performance of derivative-free optimization algorithms on the same problem.

All model fitting simulations for this study were performed on the CPU partition of the high-performance supercomputing cluster JURECA^66^ at Forschungszentrum Jülich. Each computation node featured two Intel Xeon E5-2680 v3 12-core Haswell CPUs, and each core supported two hardware threads, so that one computation node (consisting of two 12-core CPUs) ultimately offered 2 × 2 × 12 = 48 threads for simultaneous computations. The base frequency of the CPUs was 2.5 GHz and the maximum memory bandwidth of one node was 136 GB/s. We refer to the work of Krause and Thörnig^66^ for further details about the configuration of JURECA.

### Grid search

At first, we considered a two-dimensional (2Dim) parameter space in which the noise intensity was fixed at *σ* = 0.3. The global weights for coupling *C* $\in$ [0, 0.945] and delay *τ* $\in$ [0, 94] were tuned along a set of 64 × 48 = 3072 discrete, equidistant points. The selected parameter range for the considered model was suspected to include the optimal model parameters of the best correspondence between simulated and empirical data, which was confirmed by post-simulation analyses^22^. Furthermore, the considered grid granularity was selected as a trade-off between the computational costs and a favorable density that allowed for a close approximation of the goodness-of-fit values as confirmed in this study by comparing the fitting results with those of other optimization methods. For each tuple (*C*, *τ*), we generated simFC which we compared to the empirical data, resulting in a total of 322 560 (64 × 48 × 105) model simulations for the considered cohort of 105 subjects. One subject required approximately 100 min (≈ 1.67 h) of calculation time on a CPU node with 48 threads for parallel computations (24 cores × 1.67 h ≈ 40 core-hours). Subsequently, we performed another thorough parameter scan in which we also varied the noise intensity *σ*. This three-dimensional (3Dim) grid search was executed for 48 × 22 × 81 = 85536 tuples (*C*, *τ*, *σ*) and required approximately 1200 core-hours per subject. While the individual values for *C* $\in$ [0, 0.94] and *σ* $\in$ [0, 2] were equidistant, *τ* $\in$ [0, 48] was distributed more densely for smaller values. For a broad exploratory investigation of the noise impact, its intensity was varied between 0 (noise-free scenario) and a maximal amplitude of 2, which was inspired by Deco et al.^14^ and indicates a very strong perturbation of the system dynamics. In view of the rising computational requirements, the highest value of delay *τ* was reduced to 48, which however did not distort the results, because the parameters maximizing the model fit did not suggest very large delays^22^. The search in 3Dim comprised about 9 M model simulations for 105 subjects included in the study.

A few examples of the goal function *F* = *F*(*C*, *τ*) depending on the global coupling *C* and delay *τ* are shown in Supplementary Fig. S1, where the noise intensity is set to *σ* = 0.3 (in accordance with our definition of the 2Dim parameter space). *F* exhibits a different shape for every single subject, and thus, also the value and location of the maxima are different (dark red color). Finding the subject-specific maximum of *F* constitutes the model validation against empirical data.

In the following, we outline the investigated mathematical optimization schemes that we applied in addition to the grid search. The goal was, again, to maximize the goal function *F* for each subject in the respective (2Dim or 3Dim) parameter space. Since most methods are traditionally formulated as function minimizers, we internally worked with −*F*: a maximization of *F* is equivalent to a minimization of −*F*.

### Nelder-Mead Algorithm

The first method tested was the Nelder-Mead Algorithm (NMA), introduced more than 50 years ago by Nelder and Mead^50^. It is also known as the Downhill Simplex Method and belongs to the class of direct local search methods. Its derivative-free optimization procedure is purely deterministic, updating in each iteration the worst of the given trial solutions based on the ordering of the respective function values. More precisely, the algorithm starts with an initial set of Λ = Dim + 1 points that define the vertices of a simplex in the parameter space. In the most vivid case, Dim = 2, this corresponds to a simple triangle. After evaluating the objective function at all vertices, the algorithm replaces the vertex with the highest function value by a new point found through a series of transformations around the centroid of the remaining ones. These operations include, among others, expansions and contractions of the current simplex. Ideally, this procedure will create a sequence of simplices of decreasing size which accumulate around the desired optimum of *F*. More details on the strategy can be found in Lagarias et al.^67^ and Hicken et al.^68^ as well as in the Supplementary Materials. We also define the utilized stopping criterion (sufficiently short simplex edges), which effectively terminated the optimization before reaching the maximum of 80 iterations in more than 99% of the performed algorithm executions. A few examples of the best goal function values depending on the number of executed iterations are shown in Supplementary Fig. S2.

### Particle swarm optimization

The algorithm of Particle Swarm Optimization (PSO) was the second method in our considerations. It is a population-based variant of global stochastic search strategies and has originally been introduced by Kennedy and Eberhart^51,69^. Derived from the simulation of natural phenomena such as bird flocking or fish schooling, it features a swarm of $\Lambda \in {\mathbb{N}}$ particles (we selected Λ = 60 after an internal hyperparameter optimization) exploring the parameter space collaboratively and exchanging information about the discovered function values in each step. The individual particles are given by their coordinates in the parameter space and can be regarded as trial solutions in the iterative optimization of the goal function. They are equipped with particular velocities that are repeatedly updated and therefore enable an exploratory roaming along distinct trajectories. Throughout the iterations, every particle remembers its individual best position, i.e. the location in the parameter space where an evaluation of the objective function yielded the lowest value on the particle’s way so far. This information is shared with the entire swarm, so that the current global best position of all individuals can be determined and communicated across particles based on the respective function values. In the velocity update, each particle then adjusts its moving direction according to the distance to both its individual most satisfactory position and the global best point. The aim of this strategy is to seize the advantages of social sharing of information during a collaborative search. Individual best points lying close to the global one, eventually lead to a dense swarm with particles gathered around the suspected global optimum of *F*. More detailed outlines of the algorithm can be found in related mathematical works^70–72^ and in the Supplementary Materials. In roughly 11% of the executions we performed, the optimization was terminated prematurely due to a saturation and lack of improvement during 50 (out of 80) consecutive iterations. A few examples of the convergence and the optimal *F*-values depending on the iteration steps are illustrated in Supplementary Fig. S2.

### Covariance matrix adaptation evolution strategy

The third algorithm under our investigation was the Covariance Matrix Adaptation Evolution Strategy (CMAES). Since its introduction^52^ and subsequent concretization^53^, it has proven robust and successful in many black-box optimization problems^73–75^. CMAES shares PSO’s feature of being a population-based optimization approach. In the evolution strategy, however, the underlying concept is different, and particles do not roam the parameter space on individual trajectories. Instead, the points from every iteration are regarded as a *generation* from which only the best members are selected to form the population for the next step. The optimization procedure starts with a particle generation of moderate size (default $\Lambda = 4 + \lfloor 3 \ln ( \text{Dim} ) \rfloor$)^76^, which may be increased. We selected Λ = 24 after an extensive algorithm testing. Then, it works with a weighted mean of $\lfloor \Lambda / 2 \rfloor$ solutions which yield the lowest values of the goal function. Afterwards, a new generation of search points is obtained by taking Λ samples from a multivariate normal distribution centered around the weighted mean of these most promising points. The (co)variance is determined by a matrix whose update formulas take the location of the current best points into account. In this way, the search distribution is adapted iteratively towards a concentration around the optimal solutions, see Hansen et al.^76,77^ and the Supplementary Materials for further details. An early termination due to saturation and lack of improvement of the goal function during 50 (out of 80) consecutive iterations was observed in approximately 24% of the executions we performed. A few examples of the convergence and optimal *F*-values generated by the algorithm after every iteration step are presented in Supplementary Fig. S2 for a few subjects.

### Bayesian optimization

Lastly, we also considered the strategy of Bayesian Optimization (BO). Some of its conceptual principles can already be found in early works^78,79^. However, the current form of BO as a sequential design strategy for global optimizations of black-box functions has been discussed in more recent papers^54,80^. Starting with a random sample of a small to moderate number of candidate solutions (Λ ≤ 10 Dim, we used Λ = 5 to 10) in the parameter space, BO works with a probabilistic surrogate model for the goal function. Typically, *Gaussian Process Regression*^81^ is applied. The model is adjusted based on every new function evaluation, and further sampling points are found iteratively by an *acquisition function*. It indicates the area where the highest gain can be expected. One advantage of this algorithm is that it incorporates the information from all previous evaluations in order to estimate the shape of *F*. Additionally, the internal procedures can be adjusted especially for the case of function values perturbed by noise. Detailed descriptions of the individual steps can be found in algorithm-related works^82,83^ and in the Supplementary Materials. In our simulations, we used the C++ software package *BayesOpt*^84^. It allowed for the utilization of the stopping criterion in the form of a maximal number of iterations, which we set to 80. A few examples of the method convergence and the optimal values of the goal function depending on the number of executed iterations are shown in Supplementary Fig. S2.

### Algorithm executions and success probability

For all tested subjects, we first executed each method for *R*_max_ = 15 times with different random initial data in the respective parameter spaces. From the individual runs, we extracted the highest model fitting value and the corresponding parameter constellation. The values for optimization were bounded to (*C*, *τ*) $\in$ [0, 1] × [0, 100] in the 2Dim scenario and to (*C*, *τ*, *σ*) $\in$ [0, 1] × [0, 100] × [0, 2] in the 3Dim case.

Then, we also investigated how the number of algorithm executions with different initial data can influence the model validation results. We therefore evaluated how many runs of a given optimization method are on average necessary to obtain a goodness-of-fit of at least 95% of that from the grid search. In cases where the quality of the model validation of the grid search was not reached by a given algorithm after the default number of *R*_max_ = 15 runs, we performed some additional simulations to test whether a larger number of algorithm executions can improve the model fitting. This resulted in a total of *R*_max_ = 24 executions for NMA and BO, see “Results”.

To calculate a probability of reaching 95% of the goodness-of-fit of the grid search for a given number of runs, we started with randomly selecting *R* = 1, 2, …, *R*_max_ goodness-of-fit values out of the available solutions of a given algorithm. After comparing the selected values to the threshold, we assigned either 0 (for failure, i.e. the grid search result was not reached by any of the selected values) or 1 (for success, i.e. grid search result was reached by at least one of the selected values) to the current set of *R* goodness-of-fit values. This procedure was repeated 500 times for every choice of *R*, subject and optimization algorithm. For every method, we then computed the mean success rate (from all 500 trials) for each subject and then averaged it over all subjects for every value of *R.* This gave us a subject-independent estimate of the probability to reach the threshold with *R* algorithm runs. We henceforth refer to this quantity as a *success probability*. In such a way we evaluated the expected number of optimization runs needed by every considered method to reach a certain success probability of the model fitting quality comparable with the grid search results. This number of runs played a crucial role in comparing the actual time requirements of the individual algorithms, where the computational resources were calculated for the number of runs needed to reach the same success probability.

### Cost function $\Psi$_cost_

For a systematic evaluation and comparison of the tested optimization algorithms, we defined a cost function $\Psi$_cost_ that combines several individual properties of the algorithms including the goodness-of-fit, the computation time and the location of the optimized parameters that can be used to assess the quality of the optimization. The components of the cost function represent the features along which we estimated every method’s advantages and limitations. In this procedure, we assigned each algorithm a certain cost value for every subject and interpreted lower costs as better results.

To construct $\Psi$_cost_, we specify the notation that we will use in the following. For each subject and algorithm, we calculated a vector $${\text{Fit}} = \left( {\begin{array}{*{20}c} {{\text{Fit}}_{1} } \\ \vdots \\ {{\text{Fit}}_{15} } \\ \end{array} } \right) \in {\mathbb{R}}^{15}$$ collecting the goodness-of-fit values from all 15 executions, a matrix

3 $${\text{Points}} = \left( {\begin{array}{*{20}c} {P_{1} } \\ \vdots \\ {P_{15} } \\ \end{array} } \right) = \left( {\begin{array}{*{20}c} {C_{1} } & {\tau_{1} } & {\sigma_{1} } \\ \vdots & \vdots & \vdots \\ {C_{15} } & {\tau_{15} } & {\sigma_{15} } \\ \end{array} } \right) \in {\mathbb{R}}^{{15 \times {\text{Dim}}}}$$

of dimension 15 × Dim containing the optimal values of the respective model parameters (*C*, *τ*, *σ*), and a scalar ${\text{Time}} \in {\mathbb{R}}$ representing the cumulated computation time required for executing the number of algorithm runs needed to obtain a success probability of 80% (see previous section). In the 2Dim case, the third column with the noise intensity *σ* disappears in the matrix Points.

For the grid search in turn, we extracted a matrix

4 $${\text{GridPoints}} = \left( {\begin{array}{*{20}c} {G_{1} } \\ \vdots \\ {G_{5} } \\ \end{array} } \right) = \left( {\begin{array}{*{20}c} {C_{1}^{GS} } & {\tau_{1}^{GS} } & {\sigma_{1}^{GS} } \\ \vdots & \vdots & \vdots \\ {C_{5}^{GS} } & {\tau_{5}^{GS} } & {\sigma_{5}^{GS} } \\ \end{array} } \right) \in {\mathbb{R}}^{{5 \times {\text{Dim}}}}$$

of dimension 5 × Dim that contains the parameter values corresponding to the five highest correlations between simFC and empFC discovered in the exhaustive parameter space scan. Again, the last column listing the noise intensities was removed in the 2Dim case. The cost function $\Psi$_cost_ can then be constructed from its individual components as explained below.

1. The first factor considers the highest goodness-of-fit value that a particular algorithm discovered based on its 15 executions. Since the model fit is measured by the Pearson correlation coefficient between empFC and simFC, this quantity is limited to the range [− 1, 1]. Then the simple difference 1 − max{Fit_*i*_}, 1 ≤ *i* ≤ 15, reflects higher Fit values as lower costs.
2. For the second factor, we took the spread of the objective function values (i.e. the detected goodness-of-fit values) into account. We therefore computed the standard deviation (SD) of the 15 model fits for every algorithm and subject. This quantity assigns higher costs to methods that tend to converge to local maxima besides the global ones, reflected by a higher variance in the respective goodness-of-fit values.
3. The third component incorporates the computation time required for the number of algorithm executions necessary to reach a success probability of 80% (see previous section).
4. As the next important ingredient of $\Psi$_cost_, we considered the spread of the optimized parameters across replications. We therefore computed the mean Euclidean distance between the 15 solutions of a given algorithm in the parameter space (normalized to [0, 1]^Dim^). In this setting, a lower spread indicates a higher stability of the respective method, while larger values hint at a convergence to more than one (local) optimum.
5. Finally, the distance of the algorithm solutions to the optima found by the grid search plays a role in the cost function. For the latter method, we extracted five parameter points per subject (GridPoints) that provided the highest goodness-of-fit values and which approximated the global optimum, as mentioned above. We then computed the average pairwise Euclidean distances between the 15 algorithm solutions on one side and the five grid search solutions on the other side. This term was included in the cost function in order to evaluate how much the calculated algorithm solutions deviate from the approximate ground truth given by the results of the grid search. Additionally, we aimed at making more precise differentiations in cases where the algorithms converge to more than one solution.

For better interpretability, the calculated values were normalized to the interval [0, 1] by dividing by the corresponding maxima (calculated over all algorithms for a given component in the respective parameter space).

We multiplied the mentioned components in order to obtain the final values of $\Psi$_cost_ for each algorithm and subject. In summary, we obtained a 105 × 4 matrix (subjects × algorithms) containing all values. The complete form of the cost function is the following:

5 $$\begin{aligned} \Psi_{{{\text{cost}}}} & = \left( {1 - \mathop {\max }\limits_{i} \left\{ {{\text{Fit}}_{i} } \right\}} \right) \cdot \left( {\sqrt {\frac{1}{15}\mathop \sum \limits_{i} \left( {{\text{Fit}}_{i} - \frac{1}{15}\mathop \sum \limits_{i} {\text{Fit}}_{i} } \right)^{2} } } \right) \\ & \quad\cdot {\text{Time}} \cdot \left( {\frac{1}{105}\mathop \sum \limits_{i > j} \| P_{i} - P_{j}\|_{2} } \right) \\ &\quad \cdot \left( {\frac{1}{15}\mathop \sum \limits_{i} \mathop {\min }\limits_{k} \|P_{i} - G_{k}\|_{2} } \right). \\ \end{aligned}$$

In addition to the mere distribution of costs, we also evaluated for how many subjects a given method performed better than the others. To compute this quantity, we compared the costs of all algorithms for every subject individually. In other words, we sorted the cost function values assigned to the four methods (NMA, PSO, CMAES and BO) in ascending order and selected the algorithm featuring the minimal value as the favorable method for the particular subject. Repeating this procedure for the entire cohort yielded a vector of 105 subject-specific algorithm recommendations. The method gathering most of the recommendations (subjects) can be regarded as the *winner of the competition*.

## Results

To explain and analyze the characteristics of the investigated optimization methods, we will pursue the following presentation flow. We start with an illustration of their performance for one subject, where we briefly address the three evaluation criteria (see Sect. “Overview” in “Methods”) along which the individual methods may differ from each other.

Then, we turn to analyzing the algorithms more systematically based on their results across all subjects. These investigations will be guided by the three criteria from above, too. With the help of the cost function $\Psi$_cost_, we ultimately take all methods’ particular advantages and limitations into account and identify the most favorable approaches.

### Examples of the model validation for one subject

**Figure 1:**
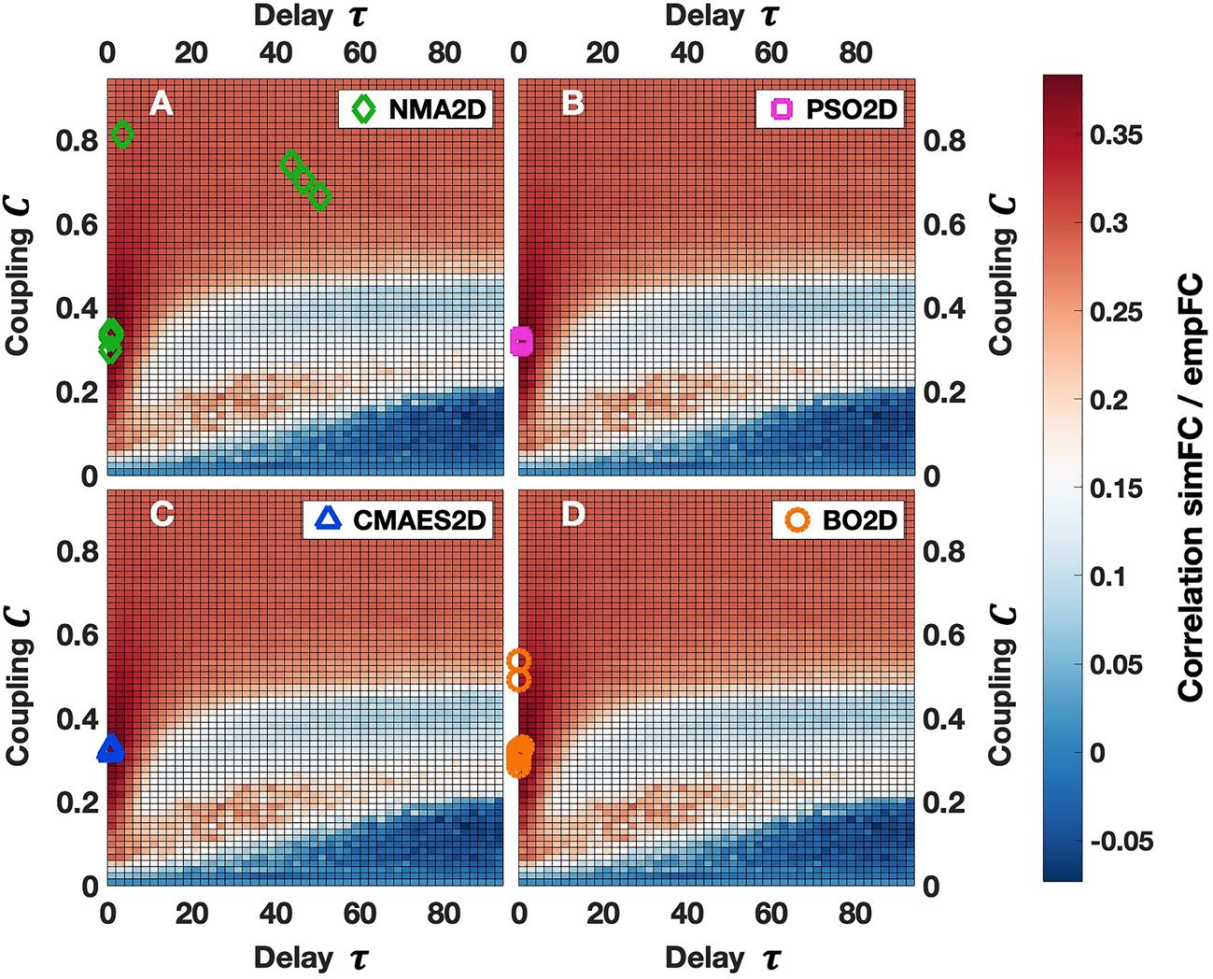
Examples of the algorithms’ solutions in the 2Dim parameter space. All plots illustrate the outcome for the same subject. As indicated in the legends, the results of the 2Dim parameter optimizations via NMA (NMA2D), PSO (PSO2D), CMAES (CMAES2D) and BO (BO2D) are visualized by colored symbols (diamonds, squares, triangles and circles, respectively). For each method, 15 optimal parameter points obtained from 15 algorithm executions with random initial conditions are indicated. The background fill of the plots illustrates the shape of the parameter space as discovered by the grid search, see also Fig. S1. It shows the similarity (Pearson correlation) between simulated and empirical FC on a color scale reaching from dark blue (low similarity) to dark red (high similarity). The figure was created with MATLAB R2018a (www.mathworks.com).

In the illustrative example in Fig. 1, we observe that all methods are able to find the area of the highest similarity (global optimum) between simulated and empirical FC, which can be verified by the results of the grid search (dark red color). The highest values (out of 15 runs) of the detected goodness-of-fit and the corresponding optimal parameters may, however, vary to small extent across the considered methods (Table 1). A possible cause of this effect may be the stochastic impact of the noise included in the model that influences the results. Most methods in the considered example outperform the grid search slightly (between 0.3% and 2.5%) with respect to the goodness-of-fit values, except for CMAES (− 0.7%). However, this does not necessarily imply that the global optimum in the parameter space has been missed here; the solutions are rather perturbed by noise. Therefore, the values may be somewhat below the goodness-of-fit obtained by the grid search, despite the fact that the optimal parameters have correctly been identified within the dark red region.

**Table 1:** Optimal parameter points, goodness-of-fit and the used computation time for the examples presented in Fig. 1.

	Optimal coupling *C*	Optimal delay *τ*	Goodness-of-fit	Invested core-h
GS2D	0.2850	0.0000	0.3779	0040.0
NMA2D	0.3323	0.8381	0.3789	0017.5
PSO2D	0.3134	0.8941	0.3814	1378.8
CMAES2D	0.3283	0.9082	0.3753	0395.9
BO2D	0.3072	0.0070	0.3873	0015.1

The indicated parameter values (coupling and delay) and the goodness-of-fit pertain to the best solution (highest goodness-of-fit) found across all 15 algorithm runs for the four optimization methods (NMA2D, PSO2D, CMEAES2D, BO2D). For the grid search in 2Dim (GS2D), the best parameter constellation obtained from a single parameter space scan is shown. The invested core-hours (core-h) for GS2D and the optimization methods represent the required resources for one thorough scan and all 15 runs in total, respectively.

**Figure 2:**
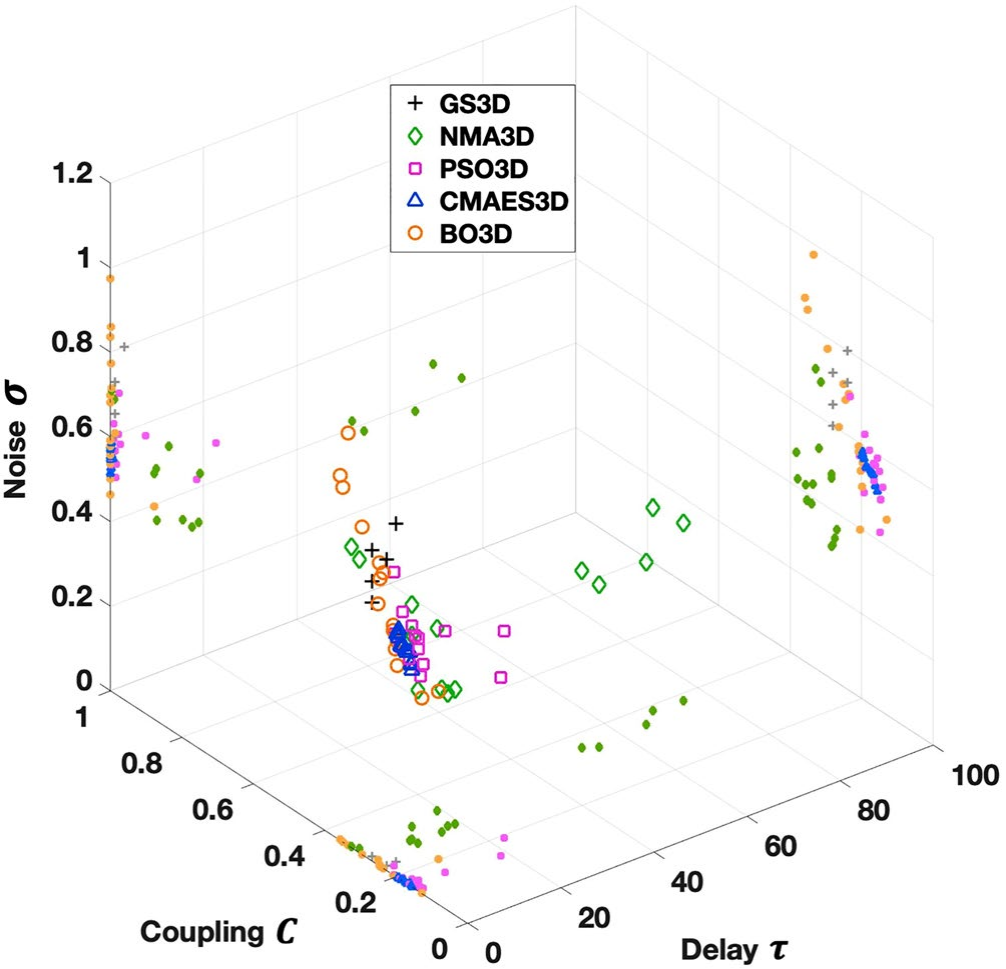
Exemplary parameter optimization in the 3Dim case. The outcomes (optimal parameter points) for all tested methods are combined in one plot. Besides the solutions in the 3Dim parameter space (larger markers), projections to 2Dim plains are presented (smaller markers). For the grid search in 3Dim (GS3D), five parameter points are considered (black crosses). In terms of the model similarity to empirical data, these points represent the five best solutions out of all values calculated on the parameter grid. As indicated in the legend, the results of the 3Dim parameter optimizations via NMA (NMA3D), PSO (PSO3D), CMAES (CMAES3D) and BO (BO3D) are visualized by colored diamonds, squares, triangles and circles, respectively. For each algorithm, 15 markers are shown (plus projections) which indicate the solutions of 15 runs executed at random initial conditions. The figure was created with MATLAB R2018a (www.mathworks.com).

For the 3Dim case, we refrained from showing the complete outcome of the grid search for illustrative purposes, but rather included only the five best points that yielded the highest fitting according to the exhaustive parameter space scan (Fig. 2). For the other optimization algorithms, the highest detected goodness-of-fit values again fluctuate around the result of the grid search (Table 2). In this example, PSO and CMAES outperform the exhaustive scan (up to 5.4%), whereas NMA and BO generate slightly lower values (between − 0.4% and − 4.1%).

**Table 2:** Optimal parameter points, goodness-of-fit and the used computation time for the results presented in Fig. 2.

	Optimal coupling *C*	Optimal delay *τ*	Optimal noise *σ*	Goodness-of-fit	Invested core-h
GS3D	0.2400	3.0000	0.8000	0.3310	1200.0
NMA3D	0.3287	0.2883	0.7079	0.3173	0020.2
PSO3D	0.1603	7.5195	0.5717	0.3489	1382.5
CMAES3D	0.1603	0.1554	0.5230	0.3479	0303.3
BO3D	0.2358	0.0042	0.6991	0.3298	0016.4

The indicated parameter values (coupling, delay and noise) and the goodness-of-fit pertain to the best solution (highest goodness-of-fit) found across all 15 algorithm runs for the four optimization methods (NMA3D, PSO3D, CMAES3D, BO3D). For the grid search in 3Dim (GS3D), the best parameter constellation obtained from a single parameter space scan is shown. The invested core-hours (core-h) for GS3D and the optimization methods represent the required resources for one thorough scan and all 15 runs in total, respectively.

Importantly, the invested computational resources vary greatly across the algorithms in the presented examples (Tables 1, 2). While 15 executions of either PSO (3447.0%) or CMAES (989.8%) surpass the requirements of a two-dimensional grid search clearly, NMA (43.8%) and BO (37.8%) already demonstrate speedup factors higher than 2. In the 3Dim case, the gains seem to be even more pronounced. All methods except for PSO require less resources for 15 independent optimization runs than one thorough grid search. CMAES (25.3%) appears to be nearly 4 times faster, whereas NMA (1.7%) and BO (1.4%) reach speedups close to 60 and 73, respectively.

Another crucial aspect of the method performance is the algorithm’s susceptibility with respect to local optima and the spread of solutions. We tested this by restarting the optimization for *R*_max_ = 15 random initial conditions as mentioned above and highlighted the spread of the obtained solutions by 15 corresponding markers in the parameter spaces in Figs. 1 and 2 for 2Dim and 3Dim, respectively. In the considered examples, the solutions do not always aggregate in a relatively small, distinct area, which especially becomes obvious in the 3Dim case (Fig. 2; Table 2). Instead, they may be distributed across a broader region within the parameter space. NMA, as a local search method, demonstrates a large susceptibility to local optima, to give an example. For CMAES, by contrast, the solutions converged to a specific area of the parameter space, where they form a dense cluster. We discuss the stability of the considered optimization methods as well as the other comparison criteria in more detail later below.

### Group-level analysis

#### Goodness-of-fit

**Figure 3:**
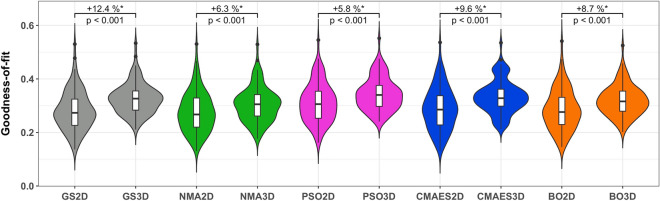
Distributions of the goodness-of-fit values for all tested approaches and dimensionalities. The considered methods and the respective parameter space’s dimension are indicated on the horizontal axis (GS2D, NMA2D, PSO2D, CMAES2D, BO2D in 2Dim and GS3D, NMA3D, PSO3D, CMAES3D, BO3D in 3Dim) along with the detected goodness-of-fit values on the vertical axis. Violins show the distributions of the highest model fit detected for all subjects. The medians (across subjects) of the relative increase between the results in 2Dim and 3Dim are indicated in the plots together with *p*-values of the Wilcoxon signed-rank test. Statistically significant differences are marked with an asterisk (the significance level of 5%, *p* < 0.05, has been Bonferroni-corrected for multiple comparisons).

**Figure 4:**
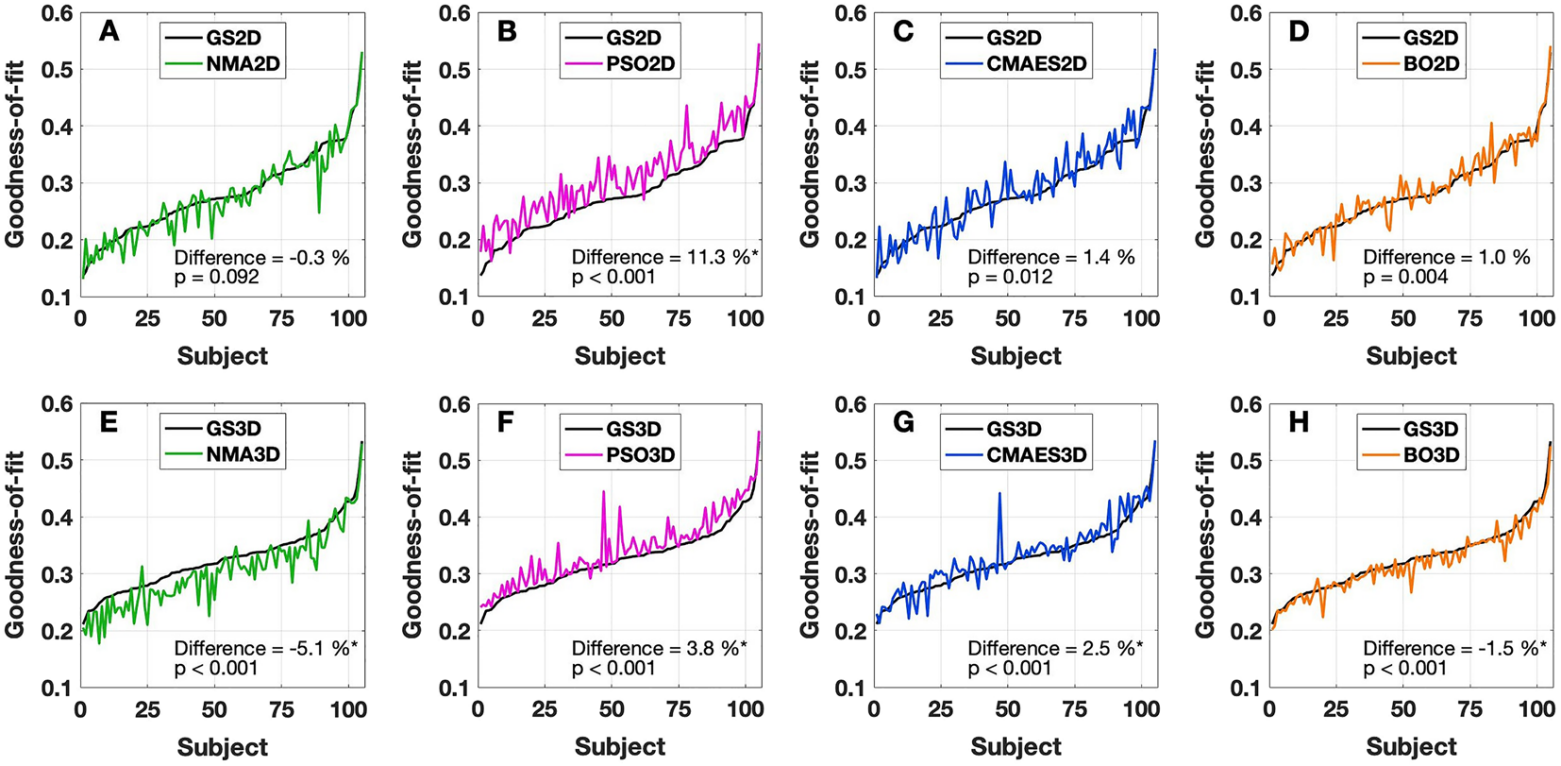
Goodness-of-fit values for individual subjects and optimization approaches. As indicated in the legends, colored curves illustrate the outcome (subject-dependent goodness-of-fit) for each method in the (**A**–**D**) 2Dim (NMA2D, PSO2D, CMAES2D, BO2D) and (**E**–**H**) 3Dim (NMA3D, PSO3D, CMAES3D, BO3D) cases. The subjects are sorted in ascending order, based on the maximal fitting values detected by the grid search (black curves) in 2Dim and 3Dim (GS2D and GS3D, respectively). The medians (across subjects) of the relative differences between the results of the optimization algorithms and the grid search are indicated in the plots together with *p*-values of the Wilcoxon signed-rank test. Statistically significant differences are marked with an asterisk (the significance level of 5%, *p* < 0.05, has been Bonferroni-corrected for multiple comparisons). The figure was created with MATLAB R2018a (www.mathworks.com).

**Figure 5:**
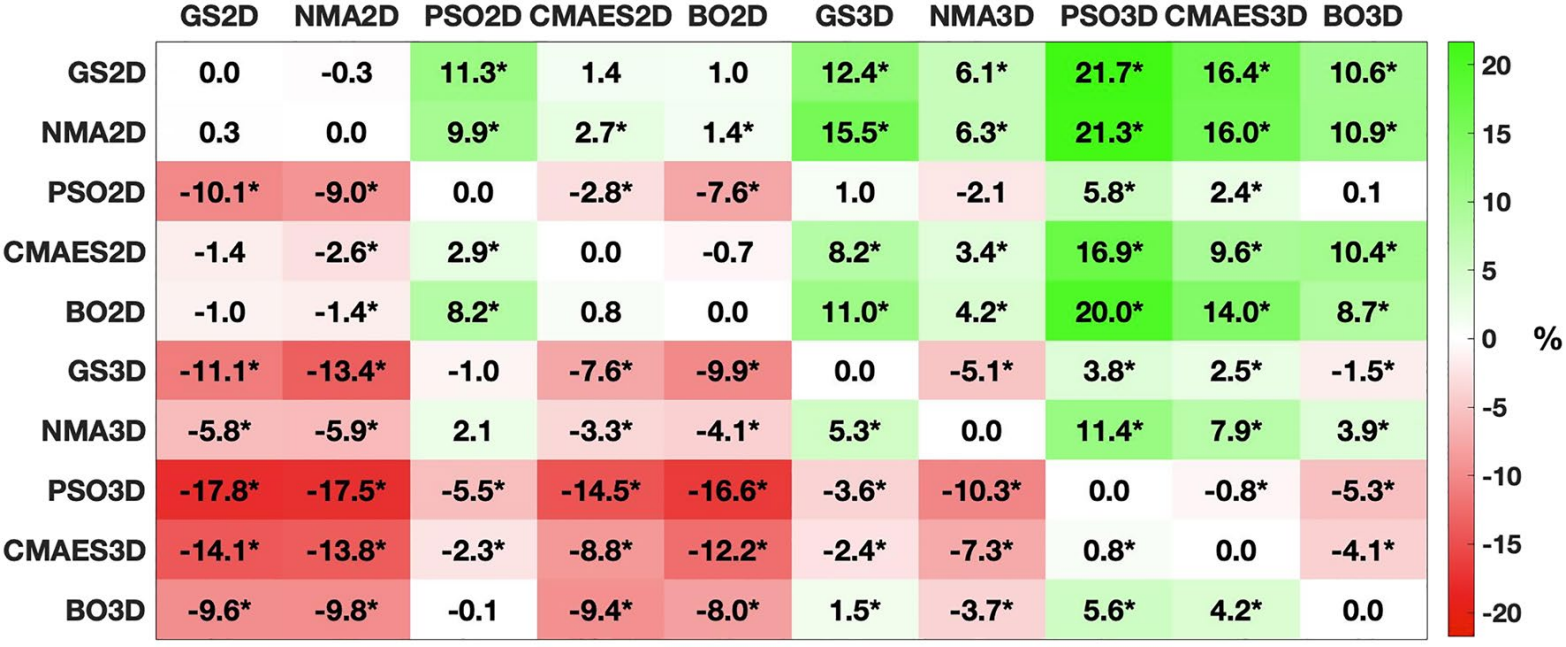
Comparison of the considered methods with regard to the detected goodness-of-fit values across all subjects. For every optimization technique and subject, the highest goodness-of-fit value is considered. The medians of the relative differences between any two methods are indicated in the cells which are highlighted in color. To this end, the goodness-of-fit values of the approaches listed on the vertical axis (lines) are subtracted from those of the methods indicated on the horizontal axis (columns). Results that proved statistically significant in the Wilcoxon signed-rank test are marked with an asterisk (the significance level of 5%, *p* < 0.05, has been Bonferroni-corrected for multiple comparisons). The figure was created with MATLAB R2018a (www.mathworks.com).

To evaluate the performance of the investigated optimization methods in more detail, we compared the detected goodness-of-fit values across all subjects. Here, we observed that the fitting quality is higher in 3Dim than in 2Dim, regardless of the applied algorithm (Fig. 3). The averaged improvement is around 8.6%. Moreover, the goodness-of-fit values obtained by the optimization methods for individual subjects can compete with those calculated by the extensive parameter optimization on a dense grid (Fig. 4). The medians of the individual relative differences to the grid search solutions evaluated across subjects range from − 0.3% to + 11.3% for optimizations in the 2Dim parameter space and from − 5.1% to + 3.8% in the 3Dim case (Fig. 4). Among the methods, the order NMA < BO < CMAES < PSO can be observed (Fig. 5).

**Figure 6:**
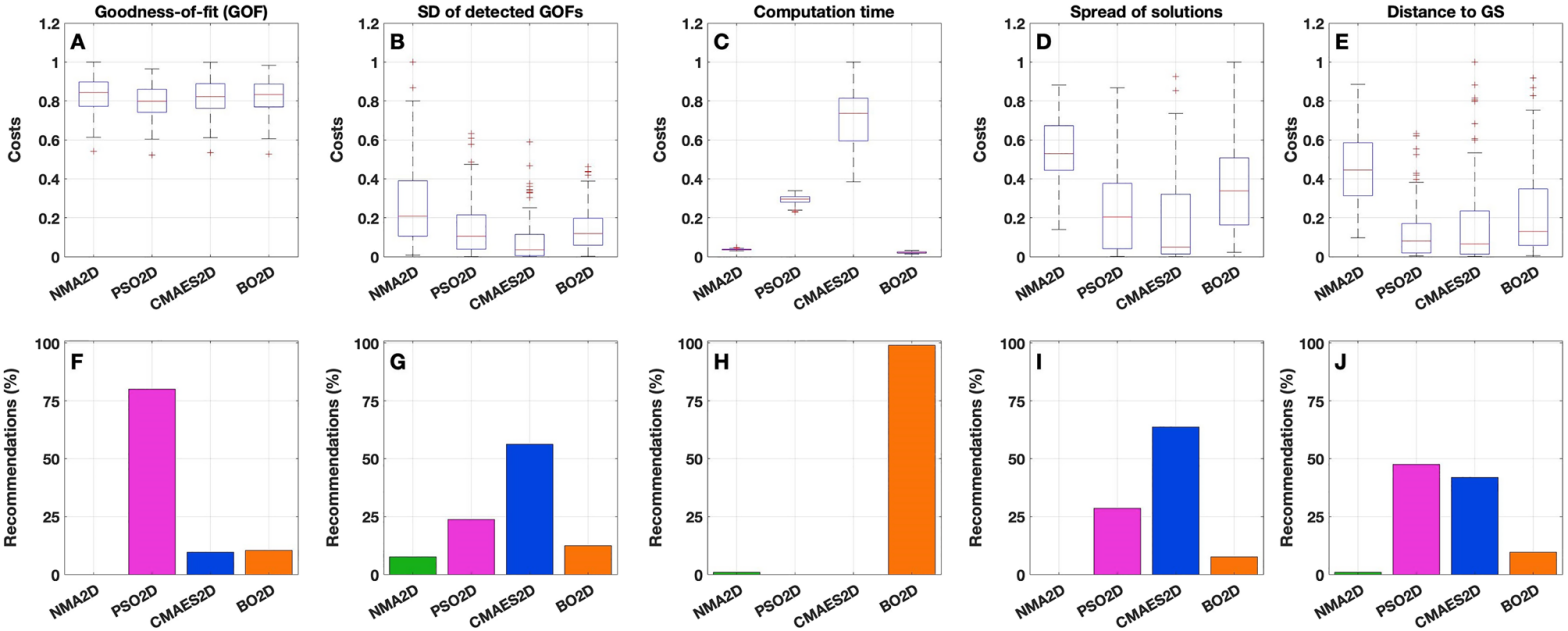
Components of the cost function $\Psi$_cost_ for the 2Dim case. (**A**–**E**) Boxplots illustrate the distributions of the values of the components indicated in the titles (see text for details). All values were normalized by the respective maxima (given as one quantity over all algorithms for a given component). The individual cost values are shown on the vertical axes while the corresponding algorithms (NMA2D, PSO2D, CMAES2D and BO2D) are indicated on the horizontal axes. (**F**–**J**) For each cost component, histograms show the relative number of recommendations obtained by a given optimization algorithm across all subjects. This quantity was calculated by counting the number of subjects for which the respective algorithm is considered *recommendable*, i.e. where it provides the lowest costs among all four investigated methods. The absolute value was then divided by the total cohort size of 105 subjects. As before, the methods are listed on the horizontal axes. On the vertical axes, it is indicated for which fraction of subjects a particular method is recommended. The figure was created with MATLAB R2018a (www.mathworks.com).

**Figure 7:**
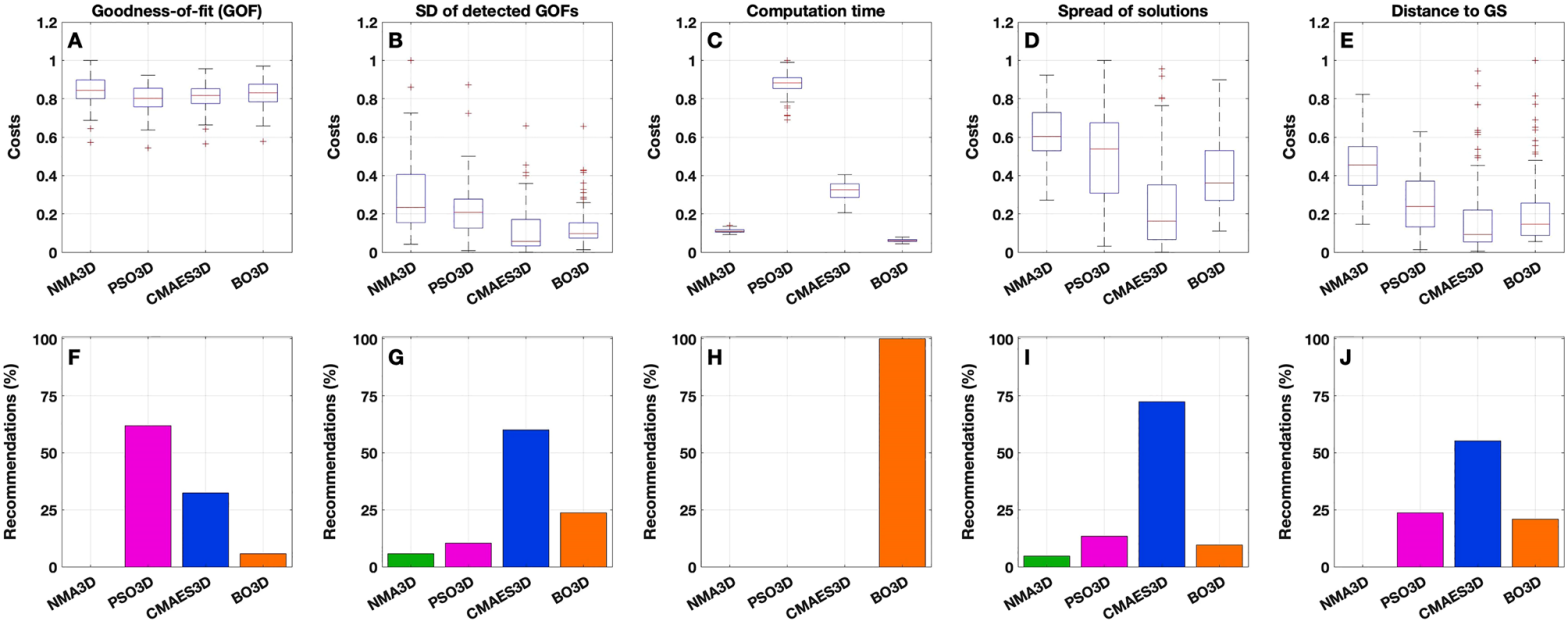
Components of the cost function $\Psi$_cost_ for the 3Dim case. As in Fig. 6, (**A**–**E**) boxplots illustrate the distributions of the values (normalized by the respective maxima) of the components indicated in the titles, and (**F**–**J**) histograms show the relative number of recommendations obtained by a given optimization algorithm (NMA3D, PSO3D, CMAES3D and BO3D) for each cost component. The figure was created with MATLAB R2018a (www.mathworks.com).

From the first component (goodness-of-fit) of the cost function $\Psi$_cost_, we see that the differences between the algorithms are not very pronounced in terms of the distribution of costs (Figs. 6A, 7A and, for the exact values, Supplementary Table S1). Nevertheless, PSO appears to produce the best results across all 105 subjects under investigation (Figs. 6F, 7F and Supplementary Table S1). Regarding the recommendations, it clearly dominates with 80% in the 2Dim case and the other methods cannot keep up with PSO here. In the 3Dim case, CMAES attracts slightly more than 30% of the subjects, but it still does not reach the performance of PSO (62%). It is remarkable that NMA does not even receive a single recommendation, meaning that it is always outperformed by at least one other method with respect to the detected goodness-of-fit values.

When analyzing the spread (standard deviation, SD) of the 15 model fits obtained for each subject and method, i.e. the second component of the cost function $\Psi$_cost_, we found that CMAES appears to demonstrate the best results, followed by PSO in 2Dim and by BO in 3Dim (Figs. 6B, G, 7B, G; Supplementary Table S1). NMA again reveals the poorest performance in both considered parameter spaces.

So, when compared to the other methods, PSO demonstrated an advantage in detecting the highest individual goodness-of-fit value within 15 executions. However, the solutions generated by CMAES have the lowest variation across 15 replications, indicating a stronger robustness against outliers and a higher stability of the fitting results.

### Optimizing the number of algorithm executions

We observed above that the quality of the model validation (goodness-of-fit) of the grid search may on average not be reached for some of the considered optimization algorithms even after *R*_max_ = 15 executions. This is the case for NMA in both considered dimensionalities and for BO in 3Dim (see Fig. 4). To test whether a larger number of algorithm runs can improve the model fitting, we thus performed some additional simulations for NMA and BO, leading to a total of *R*_max_ = 24 executions, which we took into account when analyzing the behavior of the success probability (see “Methods”).

**Figure 8:**
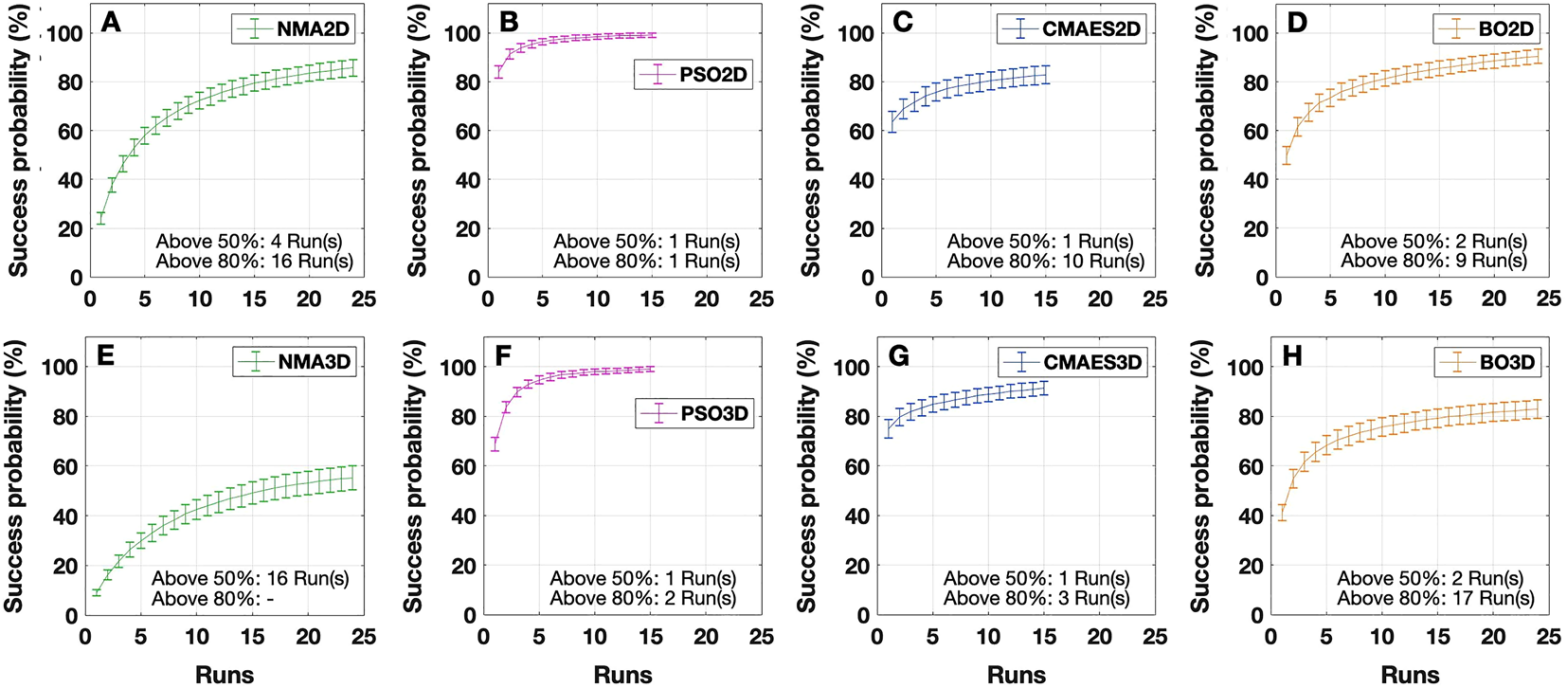
Numbers of algorithm executions and corresponding probabilities to obtain a goodness-of-fit not smaller than 95% of that from the grid search in the 2Dim (**A**–**D**) and 3Dim (**E**–**H**) cases. The probabilities were evaluated by randomly selecting $R \in \{ 1, \ldots ,R_{\max } \}$ goodness-of-fit values from the *R*_max_ algorithm executions available for every subject (*R*_max_ = 15 for PSO and CMAES, *R*_max_ = 24 for NMA and BO). A success was noted when at least one of the selected values was above or equal to the threshold. For every choice of *R* (i.e. the number of performed runs indicated on the horizontal axes), this procedure was repeated 500 times. The results were then averaged across all subjects in order to obtain the mean success probabilities indicated on the vertical axes. For the optimization methods presented in the legends (same notations as before), the plots illustrate the mentioned success probabilities together with the respective standard error (error bars). Additionally, it is indicated after how many runs success probabilities of 50% and 80% could be surpassed. The figure was created with MATLAB R2018a (www.mathworks.com).

Performing the discussed calculations, we found the following (Fig. 8): The success probability is a monotonically increasing function of the number of executed runs. The growth rate (slope of the curves), however, decays with a rising number of algorithm executions. NMA needs 4 initializations in 2Dim to reach a success probability above 50% and 16 runs to surpass 80%. PSO in turn manages to reach 80% (and 50%, of course) with only 1 initialization. For CMAES, a success probability of 50% is achieved within 1 run, compared to 10 runs for 80%. BO requires 2 executions to reach 50% and 9 to surpass a success probability of 80%. In the 3Dim case, by contrast, NMA needs at least 16 executions for 50%, but it cannot guarantee a success probability of 80% within the maximal number of *R*_max_ = 24 performed runs. PSO’s required number of runs is 1 for 50% and 2 for 80%. Interestingly, CMAES seems to perform better in 3Dim than in 2Dim. While a success probability of 50% is achieved within 1 run in either dimensionality, 3 runs already seem to suffice for 80% in 3Dim, which is much less than in 2Dim. Lastly, BO requires 2 executions to reach 50%, while 17 runs are needed to surpass a success probability of 80%.

From the success probability’s level of saturation (Fig. 8) we found that the observed success probability improvements were only marginal between 15 and 24 runs for NMA and BO. We can thus conclude that a larger number (*R*_max_ = 24) of executions did not improve the performance of these techniques considerably. In particular, we did not consider performing additional executions for PSO and CMAES and continued to focus on the standard 15 runs for all tested mathematical optimization algorithms unless otherwise indicated (needed computation time, see next paragraph).

### Computation time

**Figure 9:**
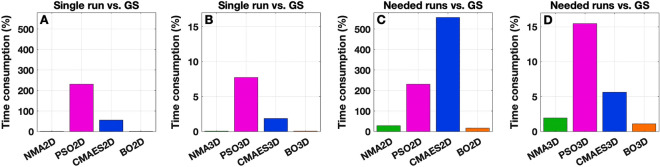
Computation time necessary for the execution of the optimization algorithms in relation to that used for the grid search (GS). (**A**, **B**) Ratios between the computation time of a single run of the optimization algorithms and the grid search averaged over all algorithm executions and subjects. The individual ratios are (**A**) 2Dim case: NMA2D 1.8%, PSO2D 231.0%, CMAES2D 55.6% and BO2D 1.9%; (**B**) 3Dim case: NMA3D 0.1%, PSO3D 7.7%, CMAES3D 1.9% and BO3D 0.1%. (**C**, **D**) Relative computation time necessary for reaching 80% of the success probability (see Fig. 8) compared to the time invested for the grid search. The values are obtained by multiplying those from the plots **A** and **B** by the respective numbers of needed runs provided in Fig. 8. For NMA3D, where a success probability of 80% could not be reached, 24 runs are considered necessary. The algorithms are indicated on the horizontal axes along with the percental values of time consumption on the vertical axes for the 2Dim (**A**, **C)** and 3Dim (**B**, **D**) cases. The figure was created with MATLAB R2018a (www.mathworks.com).

The computation time that needs to be invested in order to identify the optimal model parameters constitutes a major difference between the individual approaches (Fig. 9). Under the simulation conditions used for the grid search in 2Dim, one subject required approximately 40 core-hours of computation time to exhaustively scan the parameter space, see “Methods”. We regarded this quantity as 100% and compared the algorithms’ mean time consumption (across all executions and subjects) in 2Dim relative to it. In this respect, we observed that NMA (1.8%) and BO (1.9%) require little computation time for a single run, while CMAES (55.6%) and especially PSO (231.0%), featuring clearly more *F*-evaluations, are computationally more expensive (Fig. 9A). Therefore, the execution of 15 optimization runs for each of the latter two methods requires much more computational resources than a parameter sweep on a dense grid. A different situation was observed for the 3Dim case, where three parameters (coupling *C*, delay *τ*, noise *σ*) were optimized simultaneously. The amount of computation time necessary for the grid search drastically increases (1200 core-hours per subject, see “Methods”), whereas only a moderate change can be detected for one execution of the other investigated optimization methods. Consequently, a single run of NMA (0.1%), PSO (7.7%), CMAES (1.9%) and BO (0.1%) proves computationally inexpensive when compared to the extensive parameter space exploration. All methods except for PSO remain lucrative even if all 15 runs were necessary (Fig. 9B).

Furthermore, we investigated the algorithms’ time consumption for the number of executions that were necessary, for example, to reach a success probability of 80% (Fig. 8). We thus multiplied the number of the needed runs with the computational costs of a single run (Fig. 9). In this setting, CMAES turned out to be the most expensive method in the 2Dim parameter space, requiring more than five times (556.1%) the computational resources of the grid search (on average per subject). PSO (231.0%) is more lucrative than CMAES, but stays clearly above 100%, too. By contrast, NMA and BO require less computation time than the grid search (28.8% and 16.9%, respectively) and therefore seem favorable in this respect (Fig. 9C). They are between 3.5 (NMA) and 6 (BO) times faster than the considered grid search. In the 3Dim case, all methods manifest themselves as resource-saving alternatives to a thorough parameter space scan on a dense grid (Fig. 9D). PSO (15.5%) appears to be the method with the highest demands, followed by CMAES (5.6%), NMA (2.0%) and BO (1.1%). Equivalently, their speedup factors are close to 6, 18, 50 and 91, respectively. Note that we considered the maximum of *R*_max_ = 24 runs to be necessary for NMA, although a success probability of 80% could not be reached within 24 executions of this method. The actual amount of required computational resources for this approach may therefore be higher than indicated.

The results are further enhanced when looking at the respective component of the cost function in Figs. 6C, 7C and Supplementary Table S1, where NMA and BO clearly outperform the population-based approaches PSO and CMAES. Regarding the quantity of recommendations, BO appears to be the most favorable method in either dimensionality (Figs. 6H, 7H and Supplementary Table S1).

We summarize and conclude that BO is the preferable algorithm for a computational speedup.

### Location of optimized parameters

In the initial examples (Figs. 1, 2), we have seen that the algorithms can vary greatly regarding the spread of the detected optimal parameter points in the model parameter space. The fourth component of the cost function is dedicated to this property and therefore describes the algorithms’ stability/robustness against local optima. In our analysis, CMAES turned out to have the highest stability in detecting global solutions in either dimensionality (Figs. 6D, 7D; Supplementary Table S1) and also appeared to be the recommended method for the majority of subjects (Figs. 6I, 7I; Supplementary Table S1). This property is crucial for the reliable validation of the models against empirical data. PSO features the second lowest median of costs and the second highest proportion of recommendations in the 2Dim case. It beats NMA and BO. After the transition to the 3Dim scenario, however, the PSO algorithm does not produce as stable results as in 2Dim. BO shows a clearly lower median of costs than PSO in 3Dim (i.e. a higher stability against local optima) and comes closer to PSO’s proportion of recommendations. NMA in turn demonstrates a weakness to avoid local optima, which is reflected by the highest costs and fewest recommendations in both considered dimensionalities of the parameter space.

**Figure 10:**
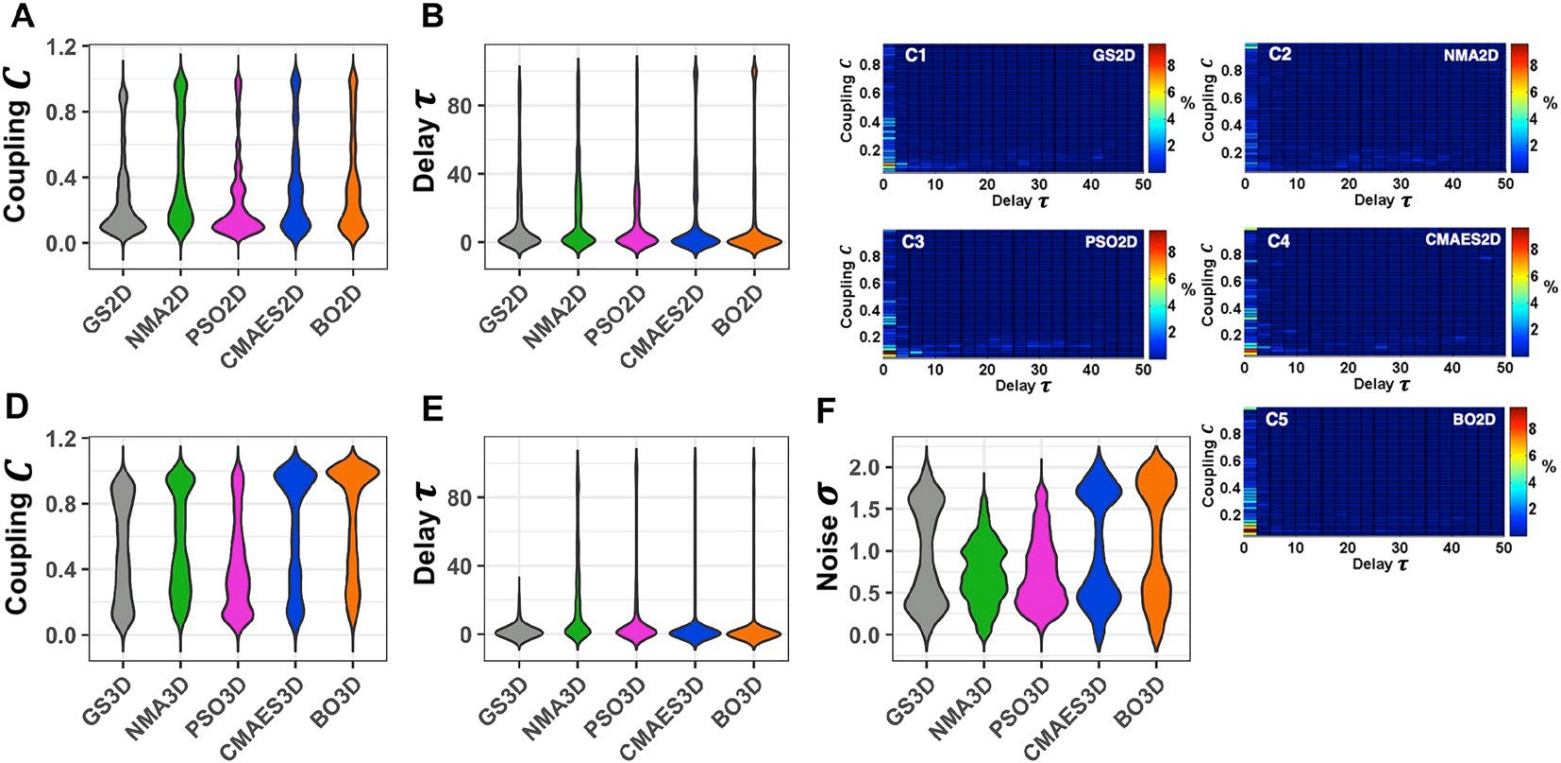
Distributions of the optimal model parameters detected by the considered techniques across all subjects. For each approach and subject, the five best solutions featuring the highest goodness-of-fit values are considered to estimate the distribution. They were selected from all similarity values computed on the 2Dim or 3Dim parameter grids (for the grid search GS2D or GS3D) and from all goodness-of-fit values obtained by 15 independent runs of the optimization algorithms (NMA2D, PSO2D, CMAES2D, BO2D in 2Dim and NMA3D, PSO3D, CMAES3D, BO3D in 3Dim). In the violin plots of one-parameter distributions for the 2Dim (**A**, **B**) and 3Dim (**D**–**F**) cases, the methods are indicated on the horizontal axes along with the optimized parameter values on the vertical axes. The plots (**C1**–**C5**) show the distributions of the optimal model parameters in the 2Dim space, where the relative frequency of the optimal parameter values is depicted by color and the respective methods are indicated in the plots. The figure was created with MATLAB R2018a (www.mathworks.com).

Investigating the distributions of the obtained optimal model parameters across all subjects, we found similarities between the considered optimization algorithms and the grid search (Fig. 10). The results presented here pertain to the approach of selecting the five best solutions featuring the highest goodness-of-fit values to estimate the parameter distributions. No qualitative changes can be noted when only one best point is considered per subject and method (see Supplementary Fig. S3). In Fig. 10, we observe that most of the methods identify the highest concentration of optimal coupling values *C* in the interval [0, 0.4] for the 2Dim case. NMA constitutes an exception, because the optimal coupling can spread to larger values for this approach (Fig. 10A, C2). A transition of the coupling strength towards an aggregation in the interval [0.8, 1] can then be observed for optimizations in the 3Dim parameter space (Fig. 10D), where also the noise intensity *σ* was varied as a free parameter. This effect is most pronounced for CMAES and BO, where skewed and nearly unimodal distributions at high coupling values can be noted. For other methods including the grid search, a broad and practically bimodal shape of the distribution of the optimal global coupling *C* emerges. In addition, all methods detect the clear majority of optimal parameters close to small (zero) delay values *τ*, regardless of whether a 2Dim or 3Dim parameter space is considered (Fig. 10B, C, E). Finally, we found that the arrangement of optimal noise intensities *σ* in 3Dim shows a bimodal pattern, mostly visible for the grid search, CMAES and BO (Fig. 10F). The peaks are located within the intervals [0.2, 0.6] (small noise) and [1.5, 1.9] (large noise).

Analyzing the discussed parameter distributions with the help of the cost function’s respective component, we found that the performance of both PSO and CMAES remains comparable in the 2Dim case, where the former gains more recommendations (Fig. 6J; Supplementary Table S1), and the latter demonstrates a lower median in the cost distributions (Fig. 6E; Supplementary Table S1). In the 3Dim case, the CMAES technique clearly outperforms the other algorithms (Fig. 7E, J; Supplementary Table S1). PSO and BO strive to be the next best method while NMA is left behind.

From the findings in this section we infer that CMAES is the most reliable method in terms of stability of the optimal parameters.

### Relationships between the cost function components

Before continuing, it is important to evaluate whether the considered components of the cost function may demonstrate a mutual interdependence. The robustness of the algorithms, for example, which is reflected by the spread of the solutions and their distance to the optima indicated by the grid search (components 4 and 5), but also the variation of the detected goodness-of-fit values (component 2) for a given subject, does not depend on the simulation time (Supplementary Fig. S4). Additionally, we did not find any strong linear relationships between the robustness of a method and its precision, i.e. the obtained goodness-of-fit (Supplementary Fig. S5). These results underline the necessity to analyze the tested algorithms from different perspectives, such as the three utilized evaluation criteria (see Sect. “Overview” in “Methods”) and the corresponding components of the cost function, before recommending a particular method. Researchers may nevertheless base their choice of algorithm on the properties best suited to a given application. One may intend, for example, to pick the method which most reliably detects the correct parameter region as the one featuring the highest model fit, irrespective of any other algorithm properties. This might then require assigning larger weights to the cost function components 4 and 5, for instance.

### Overall costs and final argumentation

**Figure 11:**
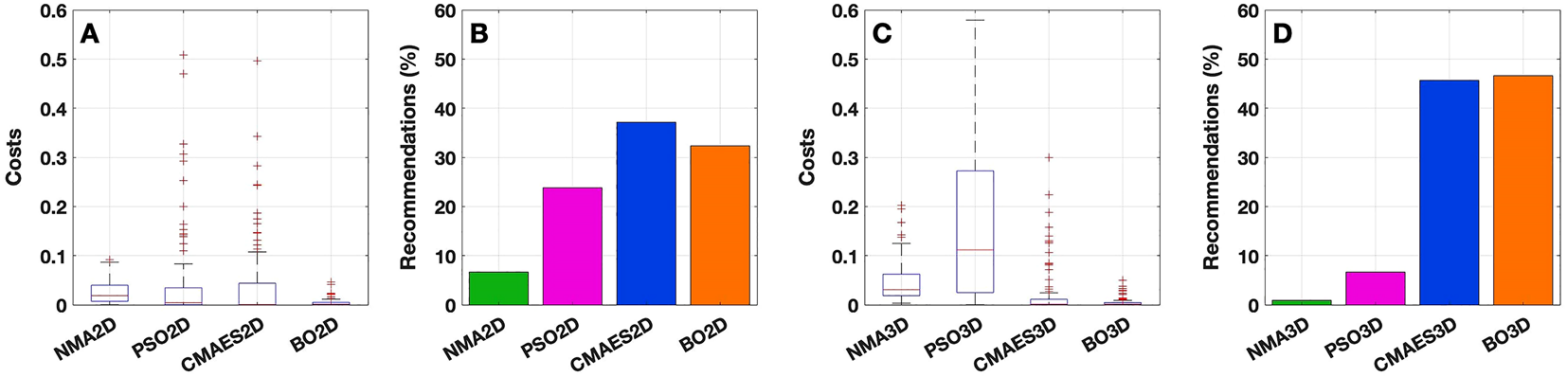
Cost function values and recommendations pertaining to the optimization algorithms for the 2Dim (**A**, **B**) and 3Dim (**C**, **D**) cases. (**A**, **C**) Boxplots illustrate the distribution of costs (normalized by the respective maxima, i.e. one value over all algorithms in the given parameter space). The considered methods are depicted on the horizontal axes together with the values of $\Psi$_cost_ on the vertical axes. A few outliers for PSO (> 0.6 in 3Dim) and CMAES (> 0.6 in 2Dim) are not included in the plots. (**B**, **D**) Histograms show the relative number of recommendations obtained by a given optimization algorithm (calculated as in Fig. 6). The methods are listed on the horizontal axes. On the vertical axes, it is indicated for which fraction of subjects a particular method is recommended. The figure was created with MATLAB R2018a (www.mathworks.com).

In this paragraph, we investigate the overall costs of the algorithms and therefore consider the complete cost function $\Psi$_cost_ which was generated by multiplying the individual components that we discussed above. The distributions of the cost function values for the tested algorithms in the considered parameter spaces are illustrated in Fig. 11. Additionally, the figure shows which methods most frequently perform better for individual subjects and can therefore be recommended for optimizations of the similarity between empirical and simulated data. The exact values are provided in Supplementary Table S1. In the 2Dim case, the algorithms show a similar distribution of low costs (Fig. 11A; Supplementary Table S1), and a precise differentiation therefore seems difficult. Based on the median cost values only, NMA appears least favorable. This is also confirmed by the proportion of recommendations calculated as explained in “Methods”, which is highest for CMAES (37%), followed by BO (32%) (Fig. 11B; Supplementary Table S1). It is remarkable that CMAES outperforms the other methods despite the fact that it features the highest computation time and corresponding costs (Figs. 6C, 9C; Supplementary Table S1). This effect may be caused by CMAES’ advantageous results for the other components of $\Psi$_cost_ (Fig. 6A, B, D, E; Supplementary Table S1). In the 3Dim case, PSO demonstrates comparably high costs, i.e. a lower efficacy (Fig. 11C; Supplementary Table S1). Unlike CMAES, the optimization quality as reflected by the components 1, 2, 4 and 5 of $\Psi$_cost_ (Fig. 7A, B, D, E; Supplementary Table S1) does not seem to be high enough for PSO to compensate for the computational resources invested for this method. PSO thus obtained fewer recommendations in the 3Dim parameter space (Fig. 11D; Supplementary Table S1). The same is true for NMA which may have the advantage of a low computation time, but does not produce as stable results as the BO algorithm, for example (Figs. 6A–E, 7A–E; Supplementary Table S1). Regarding the proportion of received recommendations in 3Dim, BO gains the most attractiveness (47%) and slightly surpasses CMAES (46%).

In summary, the mere distribution of the cost function values implies that NMA and PSO are the least favorable of the considered mathematical optimization algorithms in both the 2Dim and 3Dim parameter spaces. Further differentiations covering specific characteristics of the methods can be made either by analyzing the individual components of $\Psi$_cost_ (Figs. 6, 7; Supplementary Table S1) or by investigating the proportion of recommendations for a particular method. In the latter option, two algorithms prevalently outperform the others with their results. More precisely, for the clear majority of considered subjects, the best parameter optimization can be observed for either CMAES or BO (Fig. 11; Supplementary Table S1). This applies to both tested parameter spaces. The particular constellations of recommendations for CMAES and BO, respectively, read as follows: 37% + 32% = 69% (2Dim) and 46% + 47% = 93% (3Dim).

From the presented results we conclude that CMAES and BO are the most promising methods. CMAES turned out as the preferable approach in terms of stability, which is reflected by the low cost medians and high proportions of recommendations at the components 2, 4 and 5 of the cost function $\Psi$_cost_. Importantly, also the consideration of the computational demands, which are clearly higher for the population-based approaches PSO and CMAES (see Fig. 9), does not prevent CMAES from outperforming the other algorithms regarding the overall costs and recommendations (see Fig. 11; Supplementary Table S1). BO in turn convinces with its efficient use of resources. Its results may not be as stable as those obtained by CMAES (see the results for the components 2, 4 and 5 of $\Psi$_cost_), however, especially in the 3Dim case, they rival those of PSO. Since one execution of BO in 3Dim is performed more than 75 times faster than one of PSO, we regard BO as a more efficient method. CMAES and BO thus stand out, and we recommend to use either one or both of these mathematical schemes instead of a grid search, especially in higher dimensions (Dim ≥ 3). A thorough parameter scan on a dense grid becomes computationally unfeasible for complex models with increasingly many parameters that have to be tuned simultaneously during the process of model validation. We have demonstrated that across subjects, it is possible to obtain robust results thanks to resource-saving alternatives, i.e. mathematical optimization algorithms. Compared to a thorough parameter space scan with three free parameters, CMAES and BO provided a speedup factor close to 18 and 91, respectively. Further, the saved resources may be invested to study additional subjects in larger cohorts.

## Discussion

### Synopsis

Model-based studies should not be limited by inefficient ways to optimize model parameters. Exponentially increasing requirements in computation time, which are intrinsic to a dense grid search, triggered and motivated the search for alternatives. The intention of this study was thus to investigate the performance of several mathematical optimization algorithms in the validation of data-driven dynamical whole-brain models. We considered four well-known derivative-free methods that covered a broad spectrum of optimization techniques ranging from local deterministic (NMA) to global probabilistic (BO) and population-based (PSO, CMAES) approaches. Our results show that optimization algorithms are indeed applicable and recommendable to perform a parameter-dependent maximization of the similarity between simulated and empirical neuroimaging data. We verified this by comparing the output of the methods to the results of systematic parameter variations on a two- and three-dimensional grid. Our comparison criteria considered the obtained goodness-of-fit, the necessary computation time and the location of the optimized parameters. For the best two approaches, CMAES and BO, we obtained goodness-of-fit values being very close to the grid search solutions: The medians of relative differences across all 105 subjects ranged from − 1.5 to + 2.5% in the considered parameter spaces. At the same time, we were able to save a great extent of computational resources when optimizing three model parameters simultaneously: While CMAES already provided a speedup close to 18, BO turned out to be almost two orders of magnitude faster than a corresponding grid search. In that respect, BO demonstrated the most efficient use of resources across all tested methods. CMAES in turn produced the most stable results, which was reflected by the lowest spread of the detected parameter points and the corresponding goodness-of-fit values over 15 replications as well as by its solutions’ small distance to the optima detected by the grid search. In view of these results, further applications of the considered methods hold a lot of promise regarding the desire to facilitate the validation of higher-dimensional models.

### Contextual classification

Our efforts were in line with previous studies that analyzed and compared the performance of several mathematical methods for theoretical as well as experimental scenarios^44–46,85,86^. Similar to our strategy of incorporating the grid search to determine ground truth values, other researchers evaluated the tested algorithms’ performance on the basis of pseudo-experimental data for which the underlying parameters were known^44,46,85,86^. To our knowledge, however, no systematic comparison between the performance of mathematical optimization techniques and a grid search has been made for purely experimental data of resting-state brain dynamics measured by fMRI as in this study.

Working with electroencephalography (EEG) measurements, Hartoyo et al.^44^ and Hashemi et al.^45^ used mathematical approaches to minimize a least squares goal function that described the error between estimated and empirical power spectra. The estimated data were computed analytically and did not require the numerical solution of differential equations, therefore saving a great extent of computation time. This way of model fitting has been termed *spectral validation* by Cabral et al.^87^. The authors highlighted that this approach is less suited for obtaining an optimal quantitative fit, but should rather be used to determine a certain range of input parameters for which a model can explain the empirical data in phenomenological terms.

Our aim to maximize the correlation between simulated and empirical resting-state FC, by contrast, belongs to the so-called *spatial validation* and aligns with many previous studies^17,18,57,88,89^. Outstandingly, Wang et al.^40^ proposed an alternative to a grid search when working with resting-state fMRI data. To optimize the goodness-of-fit of a data-driven model that generated FC, the authors implemented a method derived from the expectation–maximization algorithm commonly used in dynamic causal modeling^90,91^. However, they worked with group-averaged matrices for SC as well as FC and did not perform a parameter optimization for individual subjects. A validation of the detected parameters in terms of estimating the distance to the optimal ones found in a grid search was not included, either.

A Bayesian approach closely related to the BO method which we investigated was tested for the validation of a noise-free whole-brain model that generated simulated magnetoencephalography (MEG) data^42^. The distributions of the optimized parameters from a two- and five-dimensional parameter space were thoroughly investigated, albeit without a systematic comparison to ground truth values. Further following the suggested Bayesian framework, Hashemi et al.^33^ used it to infer hidden characteristics of brain dynamics from optimized parameters in a personalized model of epilepsy spread.

Our study contributes to the existing literature by applying several mathematical optimization methods to combine a spatial validation based on simulated resting-state FC data with efforts of subject-specific whole-brain modeling. In addition, the robustness of the advocated techniques was highlighted by explicitly comparing the obtained results with the approximated ground truth given by the outcome of an exhaustive grid search. Given the topical efforts to simulate non-stationary data features such as dynamical FC, our described acceleration of computational operations appears even more welcome and relevant for the current brain research. As already envisioned by Hansen and colleagues^92^, models may be fine-tuned to not only fit static brain features, but also to account for dynamical changes in form of discrete onsets of novel behaviors, which bear an intrinsic difficulty of computing time-dependent quantities. This can be reflected by continuously changing optimal model parameters^38,93^ that need to be detected (and repeatedly updated) via efficient mathematical optimization.

### Limitations

At this point, we note that there are limitations associated with the application of the tested optimization schemes. The choice of a proper number of maximal iterations along with another adequate stopping criterion may constitute a challenge since these decisions need to be made problem-dependent. On the one hand, a low number of iterations can save precious computation time, but may not be large enough to capture an algorithm’s final point of convergence. On the other hand, a very high number of performed iterations may lead to a waste of computational resources when the algorithm is trapped in a local extremum, but may also enable a more global exploration of the parameter space. In our simulations, we chose, after an initial optimization of the methods’ internal parameters, to terminate the population-based approaches (PSO and CMAES) either after 80 iterations or if their results would not improve for 50 consecutive iterations. Stopping earlier might have been acceptable for some subjects, but not in all cases, since sudden improvements may also occur after longer periods of no gain (see Supplementary Fig. S2). Therefore, additional iterations may give the algorithms a certain amount of time to underline their potential, converge to optimal solutions and outperform the grid search. In general, we recommend to perform a series of test runs with a higher number of iterations in order to explore the characteristics and potential asymptotic behavior of a given method for a given optimization problem. Afterwards, the stopping criteria can be intensified whenever reasonable and the saved resources may be invested in additional algorithm runs with different initial data. Several executions are crucial, since the algorithms may demonstrate some sensitivity to initial data and/or do not converge to identically the same solution for different starting points. This particular issue is most pronounced for local search methods, but also applies to global approaches. In such cases, the algorithms show a high spread of solutions and fail to provide a clear tendency about the location of the global best point. A deterioration in convergence properties due to noisy measurements was reported when comparing several algorithms including PSO to each other^86^. Even global probabilistic methods turned out to be trapped in local optima mainly induced by noise fluctuations. That implies that optimization schemes can detect solutions of minor quality which deviate from the grid search result. More clearly, none of the tested algorithms provides a guarantee to find the desired optimum. Performing several runs with different initial conditions combined with an evaluation of the success probability might constitute an adequate solution to this limitation. Therefore, as presented in this study, a detailed comparison to the results of the exhaustive parameter sweep exploration is necessary to assess the methods’ performance before their application to more complex problems.

### Noise impact on the observed goodness-of-fit

As already indicated, we regard the grid search as an approximation of the ground truth (see “Methods”). However, parameter values between the grid nodes may yield slightly higher goodness-of-fit values than we observed. It is therefore not surprising that the optimization methods outperform the grid search for some subjects. The algorithms enjoy the advantage of a continuous search and do not depend on a selected grid granularity to detect optimal solutions. In addition, the methods outperforming the grid search most obviously (PSO and CMAES, see “Results”) are the population-based approaches. Here, many parameter combinations are evaluated in each execution, and only the best solutions of them were selected to determine the final goodness-of-fit values depicted in Fig. 4, for example. Repeating this procedure in presence of a random noise that ‘kicks’ the similarity between simulated and empirical data to higher values from time to time may induce a cumulative effect which leads to a somewhat higher goodness-of-fit in the end. Nevertheless, the considered parameter grid still delivers a reasonably good approximation of the ground truth.

### Outlook: personalized modeling

Given the algorithms, we see a huge potential for studies dealing with high-dimensional brain models. With the proper tools for model validation, it appears realistic to extract new and further insights about human brain dynamics from models that feature region-specific parameters or parameters associated with distinct neural populations^40,44^. Assigning a particular optimal point in the parameter space to an individual subject may be one step on the road towards possible parametrizations of inter-individual differences and a starting point for sampling individual degenerate manifolds of optimal parameters. We suppose that, in biologically motivated models, the location of optimal parameters can serve as an individual subject’s personal profile. It becomes more distinguishable from others when the underlying model simulations allow for an increased variability in form of many tunable input parameters.

The application of search algorithms might therefore play a decisive role regarding personalized modeling of brain dynamics and its implications. Thanks to the efficiently optimized demand for computational resources, further studies featuring multi-dimensional parameter space explorations on large subject cohorts may be facilitated. In this effort, the optimization procedures allow for various ways of usage. We performed up to 24 runs of each considered method in a bounded relevant parameter space and analyzed the results considering quantities such as the spread of solutions (see “Results”). Alternatively, the algorithms may also be used as a first approach to shed light into a completely unknown parameter space. A moderate number of executions could be used to determine approximate ranges or intervals of optimal parameters. These insights in turn might serve as a basis for a more precise (grid) search on an essentially smaller parameter region. In this hierarchical approach, it would be crucial to choose a method with good global convergence properties and a high stability against local optima in the first step. Afterwards, a precise strategy for the local fine-tuning of the parameters in the predetermined target region would be essential. We assume that a combination of CMAES and NMA or BO might perform well in this scenario.

## Summary and conclusions

We tested four mathematical optimization algorithms and aimed at an evaluation of the most appropriate one(s) for the fitting of dynamical whole-brain models derived from and validated against empirical neuroimaging data. To evaluate the quality of the algorithms’ output, we compared their performance on a phase oscillator model with one another as well as with a parameter space exploration on a dense grid. The results of the grid search served as an approximation of the ground truth with respect to the highest possible fit and the location of the corresponding optimal model parameters. We demonstrated that the tested methods vary greatly regarding their stability against local optima and the required computational resources. Two methods (CMAES, BO) turned out as favorable alternatives to a thorough grid search. While CMAES proved most robust in detecting global optima, BO compensated a slightly lower stability with the most efficient use of resources. We recommend CMAES and BO for optimizations starting with three free parameters, where they were around 18 and 91 times faster than a dense grid search, respectively. Our findings may contribute to a more efficient validation of complex models with high-dimensional parameter spaces and also facilitate precise and personalized modeling and analyses of human brain dynamics.

## Supplementary Information

Supplementary Information.

## Data Availability

Anonymized MRI data used for this study are available from ConnectomeDB (https://db.humanconnectome.org/). The C + + source codes of our implementations of the tested optimization algorithms are available from the corresponding author upon reasonable request.
