# Supplementary material for: Towards an efficient validation of dynamical whole-brain models
Source: Sci Rep. 2022 Mar 14;12:4331. doi: 10.1038/s41598-022-07860-7 (PMC8921267; doi:10.1038/s41598-022-07860-7)
Supplement: Supplementary file 1 — Supplementary Information. [file 41598_2022_7860_MOESM1_ESM.pdf]

Supplementary Materials

for the manuscript

Towards an efficient validation of dynamical whole-brain models

by

Kevin J. Wischnewski[1,2], Simon B. Eickhoff[1,2], Viktor K. Jirsa[3] & Oleksandr V. Popovych[1,2,*]

[1] Institute of Neuroscience and Medicine – Brain and Behaviour (INM-7), Forschungszentrum Jülich, Jülich, Germany
[2] Institute of Systems Neuroscience, Heinrich Heine University Düsseldorf, Düsseldorf, Germany
[3] Institut de Neurosciences des Systèmes (INS, UMR1106), Inserm, Aix-Marseille University, Marseille, France

[*] Corresponding author (o.popovych@fz-juelich.de)

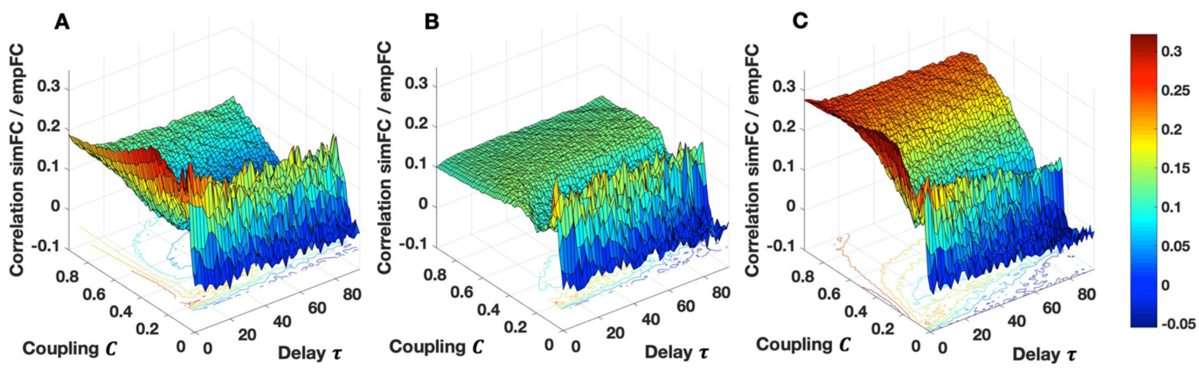

Figure S1: Examples of the goal function $F = F(C, \tau)$ for three selected subjects in the 2Dim case (with fixed noise intensity $\sigma = 0.3$). The correlation between simulated and empirical functional connectivity (simFC and empFC, respectively) is plotted versus the model parameters of global coupling C and delay τ. The values of F are also depicted by colors ranging from blue (small values) to red (large values), where the latter correspond to a better model fitting. The figure was created with MATLAB R2018a (www.mathworks.com).

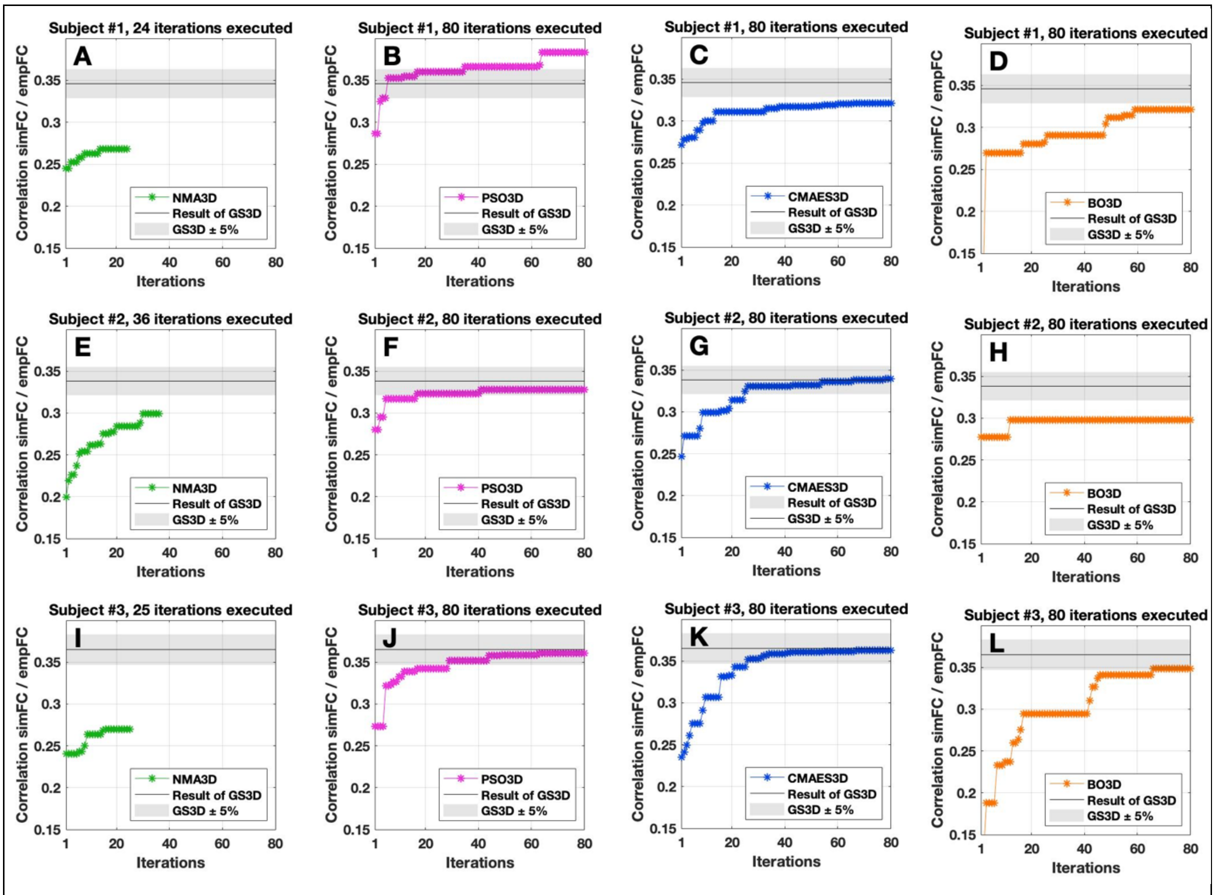

Figure S2: Examples of the algorithms' convergence as given by the values of the goodness-of-fit versus the iteration number. The plots show the gradual development of the obtained correlation corr(simFC / empFC) between simulated and empirical FC (colored lines) for one run of the methods indicated in the legends (NMA3D, PSO3D, CMAES3D and BO3D for NMA, PSO, CMAES and BO in the 3Dim parameter space, respectively). In addition to the algorithm results, the final outcome of the grid search is shown (GS3D, black line), where the shaded area represents the range from 95% to 105% of the grid search value (GS3D ± 5%). Each row represents the results for one randomly selected subject, which is mentioned in the titles along with the final number of executed iteration steps. The figure was created with MATLAB R2018a (www.mathworks.com).

	Goodness-of-fit (GOF)		SD of detected GOFs		Computation time		Spread of solutions		Distance to GS		Overall Ψ_{cost}	
	MOC	REC	MOC	REC	MOC	REC	MOC	REC	MOC	REC	MOC	REC
NMA2D	0.84	0	0.21	8	0.04	1	0.53	0	0.45	1	0.0187	7
PSO2D	**0.80**	**80**	0.11	24	0.30	0	0.20	29	0.08	**48**	0.0041	24
CMAES2D	0.82	10	**0.04**	**56**	0.74	0	**0.05**	**64**	**0.07**	42	**0.0005**	**37**
BO2D	0.83	10	0.12	12	**0.02**	**99**	0.34	8	0.13	10	0.0012	32
NMA3D	0.84	0	0.23	6	0.11	0	0.60	5	0.45	0	0.0308	1
PSO3D	**0.80**	**62**	0.21	10	0.88	0	0.54	13	0.24	24	0.1116	7
CMAES3D	0.82	32	**0.06**	**60**	0.33	0	**0.16**	**72**	**0.09**	**55**	**0.0012**	46
BO3D	0.83	6	0.10	24	**0.06**	**100**	0.36	10	0.15	21	0.0014	**47**

Table S1: Medians of costs (MOC) across subjects and proportions of recommendations (REC, in %) for the tested optimization algorithms in both the 2Dim and 3Dim parameter space. The presented values pertain to the overall cost function Ψ_{cost} as well as to its individual components (see main text for details). Bold letters indicate the most favorable numbers (i.e. lowest costs, highest proportions of recommendations) for every cost factor and dimensionality of the parameter space. The values for REC were rounded to the nearest integer.

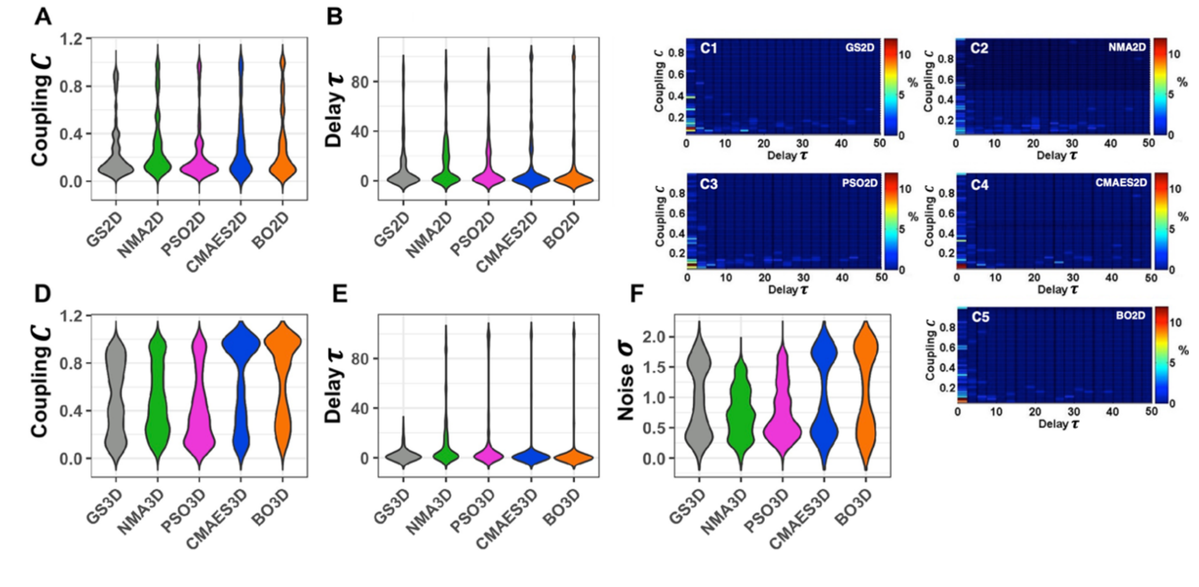

Figure S3: Distributions of the optimal model parameters detected by the considered techniques across all subjects. For each approach and subject, the best solution featuring the highest goodness-of-fit value is considered to estimate the distribution. It was selected from all similarity values computed on the 2Dim or 3Dim parameter grids (for the grid search GS2D or GS3D) and from all goodness-of-fit values obtained by 15 independent runs of the optimization algorithms (NMA2D, PSO2D, CMAES2D, BO2D in 2Dim and NMA3D, PSO3D, CMAES3D, BO3D in 3Dim). In the violin plots of one-parameter distributions for the 2Dim (A, B) and 3Dim (D-F) cases, the methods are indicated on the horizontal axes along with the optimized parameter values on the vertical axes. The plots (C1-C5) show the distributions of the optimal model parameters in the 2Dim space, where the relative frequency of the optimal parameter values is depicted by color and the respective methods are indicated in the plots. The figure was created with MATLAB R2018a (www.mathworks.com).

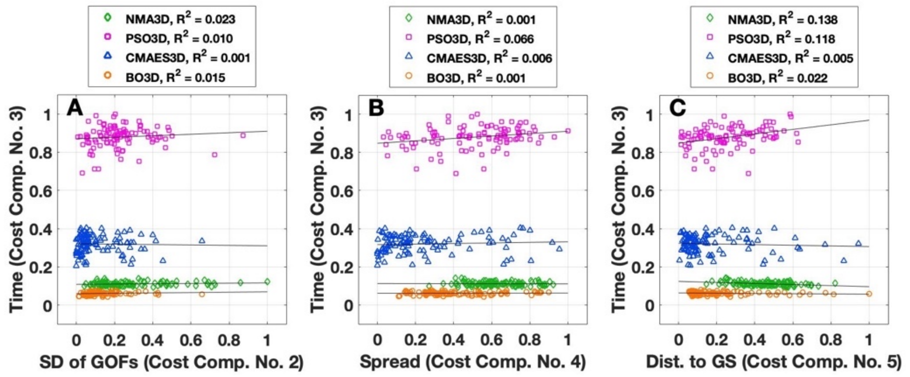

Figure S4: Relationships between the algorithms' robustness (cost function components 2, 4 and 5) and the simulation time (cost function component 3) in the 3Dim case. Scatter plots and the corresponding regression lines are shown for the normalized cost function components (Cost Comp.) describing (A) the standard deviation of the 15 detected goodness-of-fit values (SD of GOFs) per algorithm and subject, (B) the spread of the corresponding optimal parameter points and (C) their distance to the optimal points detected by the grid search (Dist. to GS) versus the required computation time (see main text for details). Each marker indicates the values for one subject. The respective regression coefficients of determination are given in the legends along with the considered algorithms. The figure was created with MATLAB R2018a (www.mathworks.com).

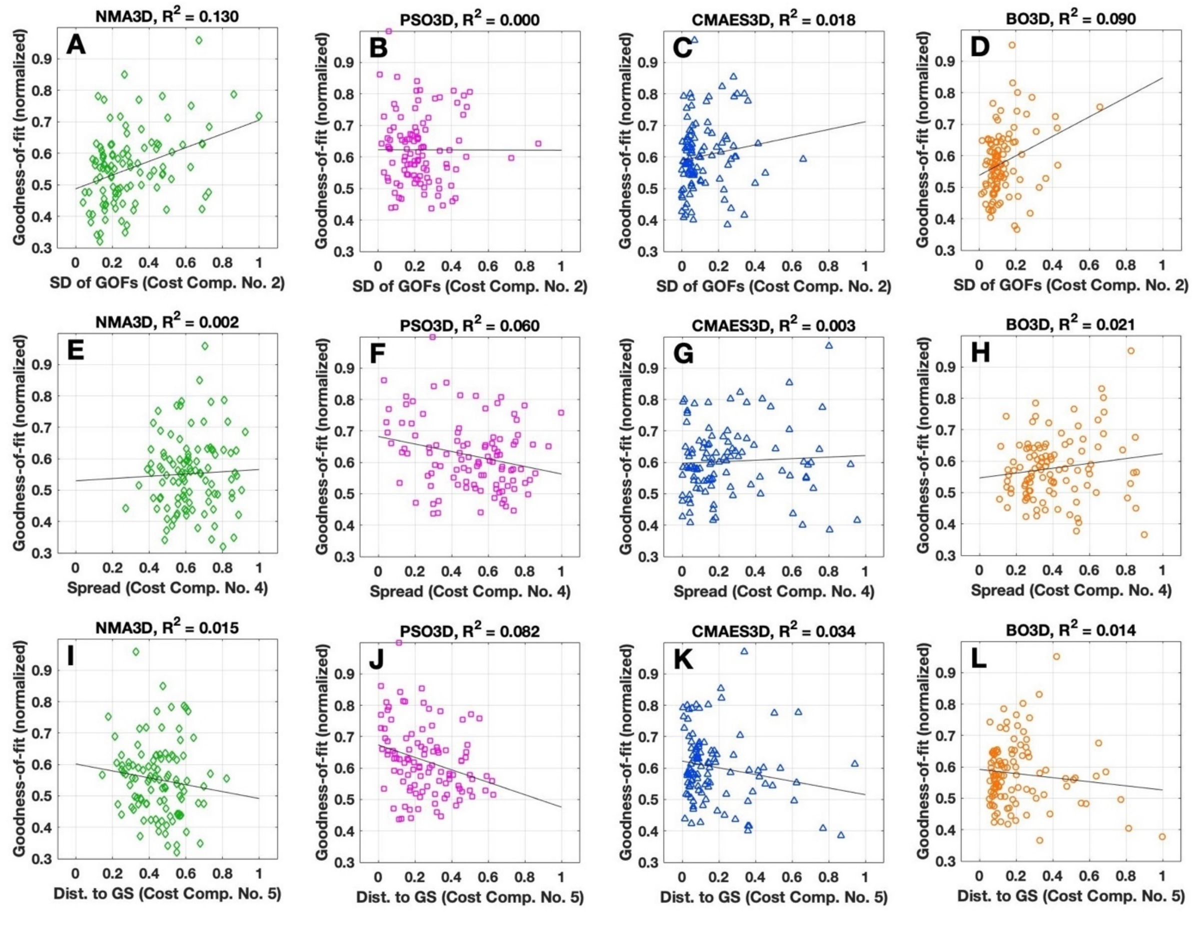

Figure S5: Relationships between the algorithms' robustness (cost function components 2, 4 and 5) and precision (goodness-of-fit derived from cost function component 1) in the 3Dim case. As in **Fig. S4**, scatter plots and the corresponding regression lines are shown for the cost function components indicated on the horizontal axes along with the normalized goodness-of-fit values on the vertical axes. Each marker indicates the values for one subject. The respective regression coefficients of determination are provided in the titles of the subplots together with the considered algorithms. The figure was created with MATLAB R2018a (www.mathworks.com).

In the following, we provide the details about the mathematical optimization schemes whose performance we discussed and compared in the presented article. The algorithms were applied to maximize the goal function F which returns the Pearson correlation coefficient between the matrices of simulated FC and empirical FC (after a vectorization of the upper triangular parts of the respective matrices).

As stated in the main text, our intended maximization of F can be formulated equivalently as a minimization of -F in order to fit to the traditional manner of describing optimization problems as minimization problems. This adaptation allowed for the usage of mathematical algorithms aiming to minimize a real-valued function $F: X \to \mathbb{R}$ defined on a parameter space $X \subseteq \mathbb{R}^{\mathrm{Dim}}$ with Dim $\in \mathbb{N}$.

Nelder-Mead Algorithm

Following Hicken et al.[1], the optimization starts with an initial set of Λ = Dim+1 points $x_1, \dots, x_{\text{Dim}+1} \in X$ not lying in the same plane and therefore defining the vertices of a *simplex*. In the most vivid case, Dim = 2, a simplex corresponds to a simple triangle. Tetrahedrons (Dim = 3) and more abstract structures appear in higher dimensions.

After computing the objective function values at all initial simplex points, those are re-ordered, so that $F(x_1) \leq \ldots \leq F(x_{\text{Dim}}) \leq F(x_{\text{Dim}+1})$. We assume here that F is not constant and therefore a meaningful ordering is possible. Then, in each iteration, the algorithm replaces the worst vortex $x_{\text{Dim}+1}$ (i.e. the one with the highest function value) by a new point found through a series of transformations around the centroid of the remaining vertices $\bar{x} = \sum_{i=1}^{\text{Dim}} x_i$.

These operations are *reflection, expansion, inside contraction, outside contraction* and *shrinking*, whereas the last one is special in the sense that it does not just replace the worst point, but decreases the size of the entire simplex instead, keeping only the current best point and adding Dim new vertices. Which of the mentioned transformations will eventually determine the new simplex for the next iteration, depends on the value of the objective function F at the proposed points relative to the current ones.

Having computed $\bar{x}$, it is probable that the straight line crossing $x_{\text{Dim}+1}$ and $\bar{x}$ defines a descent direction. *Reflection* proposes a new point lying on this line, namely

$$x_r = \bar{x} + \rho(\bar{x} - x_{\text{Dim}+1}), \qquad \rho > 0.$$

Visually, it is the mirrored equivalent of $x_{\text{Dim}+1}$ if the simplex was flipped around. We replace $x_{\text{Dim}+1}$ by x_r in case the condition $F(x_1) \leq F(x_r) < F(x_{\text{Dim}})$ is fulfilled, meaning that we obtained an amelioration compared to $F(x_{\text{Dim}})$ and $F(x_{\text{Dim}+1})$, but not yet to $F(x_1)$. If $F(x_r) < F(x_1)$, then this search direction seems to be particularly promising and we investigate the objective function value further on this line, at the *expanded* point

$$x_e = \bar{x} + \rho\chi(\bar{x} - x_{\text{Dim}+1}), \qquad \chi > \rho.$$

If $F(x_e) < F(x_r)$, then $x_{\text{Dim}+1}$ is replaced by x_e, otherwise x_r becomes the new vertex of the simplex. In case the search direction from above leads to no immediate satisfactory improvement, i.e. $F(x_r) > F(x_{\text{Dim}})$, we check whether a better point can be found by *contractions*. If even $F(x_r) \geq F(x_{\text{Dim}+1})$, we look inside the current simplex, between $x_{\text{Dim}+1}$ and $\bar{x}$, and perform an *inside contraction*, leading to

$$x_{ic} = \bar{x} + \gamma(x_{\text{Dim}+1} - \bar{x}), \qquad 0 < \gamma < 1,$$

which will be accepted if it is better than $x_{\text{Dim}+1}$, i.e. $F(x_{ic}) < F(x_{\text{Dim}+1})$. Should the condition for an *inside contraction* not be fulfilled, in other words $F(x_r) < F(x_{\text{Dim}+1})$, then an *outside contraction* with

$$x_{oc} = \bar{x} + \gamma(x_r - \bar{x})$$

is tested. x_{oc} replaces $x_{\text{Dim}+1}$ if $F(x_{oc}) \leq F(x_r)$ is satisfied.

Following these steps, it may still happen that no replacement for the worst point $x_{\text{Dim}+1}$ has been found. In that case, the Nelder-Mead Algorithm shrinks its current simplex and defines the vertices of the new one by x_1 and

$$y_i = x_i + \kappa(x_i - x_1), \qquad 0 < \kappa < 1,$$

for $i \in \{2, \ldots, \text{Dim}+1\}$. Ideally, this procedure will create a sequence of simplices of decreasing size which accumulate around the searched-for minimum of F.

For this method, we did not perform an internal parameter tuning and chose the default values[1-3]

$$\rho = 1, \chi = 2, \gamma = \frac{1}{2} \text{ and } \kappa = \frac{1}{2}.$$

The construction of the initial simplex was based on previously published ideas[4-6]. Starting with a point x_1 sampled from a uniform distribution over the parameter space, we defined a scaling value

$$c_s = \max\{1, \|x_1\|_2\}$$

together with two helper variables,

$$\beta_1 = \frac{c_s}{\sqrt{2}\mathrm{Dim}}\left(\sqrt{\mathrm{Dim}+1} + \mathrm{Dim} - 1\right) \text{ and } \beta_2 = \frac{c_s}{\sqrt{2}\mathrm{Dim}}\left(\sqrt{\mathrm{Dim}+1} - 1\right),$$

in order to construct a regular simplex whose vertices are given by

$$x_i = \left(x_{i,1}, \dots, x_{i,\mathrm{Dim}}\right)^{\mathrm{T}} \text{ with } x_{i,j} = x_{1,j} + \delta_{j,i-1}\beta_1 + \left(1 - \delta_{j,i-1}\right)\beta_2,$$

where $\delta_{i,j}$ denotes the Kronecker delta and $i \in \{2, \dots, \mathrm{Dim}+1\}$. However, we modified the procedure slightly by working with $\tilde{x}_1 = 0.35\, x_1$ as starting point and $\tilde{c}_s = 0.7\, c_s$ as scaling value. The reason for this alteration was the fact that in the original setting, the majority of our initial simplexes contained only one or two feasible points within our relevant parameter space. Since we handled boundaries by assigning a high function value, $F(x_{\mathrm{unfeas}}) = 1$, to unfeasible points, as suggested in the algorithm's introductory work[3], this led to a somewhat predictable behavior of the algorithm, namely that its optimization always began with a shrinking step. We note that this way of boundary handling may be outperformed by other alternatives[7], however, having obtained nearly degenerated simplexes when testing Box's method[8] and in view of the results[7] that even transformations of the input variables may not always be the ideal way, we held on to the comparably simple penalty approach for this algorithm.
For a stopping criterion, we focused on the length of the simplex edges. If

$$\frac{1}{\max\{1, \|x_1\|_2\}} \max_i\{\|x_i - x_1\|_2\} < 0.005,$$

then the simplex is considered small enough to stop the optimization. Additionally, we defined a maximal number of 80 iterations.

Particle Swarm Optimization

To begin the iterative minimization of F, it is necessary to define a swarm[9,10] of $\Lambda \in \mathbb{N}$ particles $x_1^0, \dots, x_\Lambda^0 \in X$ equipped with initial velocities $v_1^0, \dots, v_\Lambda^0 \in X$. One way to initialize x_i^0 and v_i^0 for $i \in \{1, \dots, \Lambda\}$ is to choose them uniformly distributed within the bounded search space X; alternatives are reviewed in other works[11,12]. Subsequently, the objective function F is evaluated at each position in the swarm and L_i, $i \in \{1, \dots, \Lambda\}$, which will serve as every individual particle's current best location, is set to $L_i^0 = x_i^0$ for all $i \in \{1, \dots, \Lambda\}$, whereas the global best position of all particles is denoted by G and initialized to $G^0 = \mathrm{argmin}\{F(x_i^0)\}$. Then, in every iteration k, the quantities v_i and x_i are updated according to

$$v_i^{k+1} = w v_i^k + \phi_L r_1^k \left(L_i^k - x_i^k\right) + \phi_G r_2^k \left(G^k - x_i^k\right),$$

$$x_i^{k+1} = x_i^k + v_i^{k+1},$$

where w denotes an inertia term[13], ϕ_L, ϕ_G represent a fixed cognitive and social parameter, respectively, and r_1^k, r_2^k are chosen randomly from the interval [0, 1].
If a particle discovers a new location that is better than its current best, i.e. $F\left(x_i^{k+1}\right) < F\left(L_i^k\right)$, then an update is made and L_i^{k+1} is set to x_i^{k+1}, otherwise $L_i^{k+1} = L_i^k$.
Analogously, the best position of the swarm is updated if $F\left(L_i^{k+1}\right) < F(G^k)$ for some $i \in \{1, \ldots, \Lambda\}$. In that case, the swarm has discovered a new global best point and $G^{k+1} = L_i^{k+1}$, otherwise $G^{k+1} = G^k$. This procedure is repeated until a stopping criterion is met.

According to Kennedy and Eberhart[10], L_i corresponds conceptually to autobiographical memory, meaning that each particle remembers the most fortunate location of its own experience. The term $\phi_L r_1^k\left(L_i^k - x_i^k\right)$ in the velocity adjustment can hence be interpreted as nostalgia; an individual tends to return to the place that most satisfied it in the past. G, by contrast, is described as publicized knowledge in the sense of a group norm or social standard that individuals strive to attain, here by the part $\phi_G r_2^k\left(G^k - x_i^k\right)$ of the velocity adjustment.
Consequently, the ratio between ϕ_L and ϕ_G determines whether a particle adjusts its velocity rather based on personal or public experiences. Tuning the balance can give the algorithm a (desired) bias towards either side and therefore guides the amount of communication among particles. Additionally, the parameter w controls the velocity updates in a way that higher values for the inertia term enforce larger update steps leading to a more global exploration of the search space while lower values render the search more detailed on a smaller, local level. w is typically initialized close to 1 and may then be gradually decreased throughout the optimization[12].

In our implementation, we tested different alternatives[12] for w (constant, linear decrease, dynamical decrease) and, besides the common choice[11,14], $\phi_L = \phi_G \approx 1.496$, we also modified the ratio ϕ_L/ϕ_G by setting either value to 1 and varying the other between 1.1, 1.2 and 1.5. Additionally, we investigated 16 different swarm sizes with Λ between 8 and 144.
Based on the results for a set of random test subjects with respect to convergence speed, most favorable objective function values and the final spread of particles in the swarm (using criteria analogous to those considered for the cost function Ψ_{cost} in the main text), we finally arrived at $\Lambda = 60$ and $\phi_L = 1 < 1.5 = \phi_G$ as the internal algorithm parameters best suited for our problems. For the inertia weight w, we adapted a procedure of dynamical decrease[12], in which we initially set $w = 0.95$ and multiplied it by 0.975 if G had not improved for 5 consecutive iterations. Similarly, we also used the adaptive penalty approach presented in the same article[12], allowing us to handle unfeasible points by adding penalties 'based on the average of the objective function and the level of violation of each constraint during each iteration step'.
Even though a component-wise limitation of the particles' velocities was no longer vital due to the introduction of the inertia weight[14,15], we still prevented too large position updates by incorporating a multiplier[16] of 0.9 for velocities whose components exceeded 20% of the feasible interval length (e.g. delay $\tau \in [0, 100]$, update limited to 20) in the respective dimension of the search space X. The optimization procedure was stopped if G had not improved for 50 iterations or if a maximum of 80 iterations had been reached.

Covariance Matrix Adaptation Evolution Strategy

As a preparation for the optimization of F, a set of quantities has to be defined. Importantly, the number Λ of particles (candidate solutions to minimize F) that form a *generation* can be freely chosen, analogously to the swarm size in Particle Swarm Optimization. However, as Hansen[17] states, '[s]mall population sizes usually lead to faster convergence, large population sizes help

to avoid local optima'. A popular recommendation is therefore to set $\Lambda = 4 + 3\lfloor\ln(\mathrm{Dim})\rfloor$ at least[17,18]. Next, $\mu = \lfloor\Lambda / 2\rfloor$ is defined. It quantifies the number of particles that will later serve as a basis to compute the generation of candidate solutions for the next iteration. More precisely, the members of the new generation $k+1$ will be sampled from a multivariate normal distribution centered around a weighted mean m^k of the μ best individuals from the previous iteration k. The selection of the best individuals is based on their respective objective function values (lower = better) and the weights are given as

$$w_i = \frac{\ln(\mu+1) - \ln(i)}{\sum_{l=1}^{\mu}\big(\ln(\mu+1) - \ln(l)\big)} \quad \text{for} \quad i \in \{1, \ldots, \mu\}.$$

With this definition, it is clear that the weights sum up to 1.
The (co)variance of the considered distribution depends on a matrix $C^k \in \mathbb{R}^{\mathrm{Dim}\times\mathrm{Dim}}$ which is adapted in every iteration. For the update, the evolution path $p_\Omega^k \in \mathbb{R}^{\mathrm{Dim}}$, the conjugate evolution path $p_c^k \in \mathbb{R}^{\mathrm{Dim}}$ and the step size $\Omega^k > 0$ are computed, along with the following quantities:

$$\mu_{\mathrm{eff}} = \frac{1}{\sum_{i=1}^{\mu} w_i^2}, \quad c_\Omega = \frac{\mu_{\mathrm{eff}} + 2}{\mathrm{Dim} + \mu_{\mathrm{eff}} + 3} \quad \text{and} \quad d_\Omega = 1 + 2\max\left\{0, \sqrt{\frac{\mu_{\mathrm{eff}} - 1}{\mathrm{Dim} + 1}} - 1\right\} + c_\Omega.$$

The latter play a role in controlling the step size. Additionally, the adaptation of the covariance matrix C^k depends on

$$c_c = \frac{4}{\mathrm{Dim} + 4}, \quad \mu_{\mathrm{cov}} = \mu_{\mathrm{eff}} \quad \text{and}$$

$$c_{\mathrm{cov}} = \frac{1}{\mu_{\mathrm{cov}}} \frac{2}{\left(\mathrm{Dim} + \sqrt{2}\right)^2} + \left(1 - \frac{1}{\mu_{\mathrm{cov}}}\right)\min\left\{1, \frac{2\mu_{\mathrm{eff}} - 1}{(\mathrm{Dim} + 2)^2 + \mu_{\mathrm{eff}}}\right\}.$$

Having defined the constants, we initialize the evolution paths to $p_\Omega^0 = p_c^0 = 0 \in \mathbb{R}^{\mathrm{Dim}}$ and select $C^0 = \boldsymbol{I} \in \mathbb{R}^{\mathrm{Dim}\times\mathrm{Dim}}$ as the initial covariance matrix. Here, $\boldsymbol{I}$ denotes the identity matrix.
The step size Ω^0 and the distribution mean m^0 have to be chosen problem-dependent. If the algorithm is used to optimize a function within a normalized space $[0, 1]^{\mathrm{Dim}}$, then $\Omega^0 = 0.5$ and m^0 uniformly randomly within $[0, 1]^{\mathrm{Dim}}$ are the popular choices[18] that we also adapted. In a newer version[17], alternative definitions not only for the starting points, but also for some constants such as the weights w_i are presented. In our test runs of the algorithm, however, the older version (from 2006) yielded slightly better results, so that we held on to it and its default values.
After these preparations, the following steps are repeated for every iteration k until a stopping criterion is met. At first, a generation of candidate solutions

$$x_j^{k+1} \sim \mathcal{N}\left(m^k, (\Omega^k)^2 C^k\right), \; j \in \{1, \ldots, \Lambda\},$$

is computed according to

$$x_j^{k+1} = m^k + \Omega^k B^k D^k z_j \; \text{ with } \; z_j \sim \mathcal{N}(0, \boldsymbol{I}).$$

$\mathcal{N}$ denotes the (multivariate) normal distribution. The matrices B^k and D^k are derived from an eigenvalue decomposition of $C^k = B^k \check{D}^k (B^k)^T$, where B^k is an orthogonal matrix containing the normalized eigenvectors of C^k and $\check{D}^k$ is a diagonal matrix containing the corresponding eigenvalues. D^k is then obtained from $\check{D}^k$ by applying the square root for the entries on the diagonal, so that $C^k = B^k D^k D^k (B^k)^T$. Afterwards, the objective function F is evaluated at every x_j^{k+1}, $j \in \{1, \ldots, \Lambda\}$, and the candidate solutions are indexed in a way that

$$F\left(x_{j_1}^{k+1}\right) \leq \ldots \leq F\left(x_{j_\mu}^{k+1}\right) \leq F\left(x_{j_{\mu+1}}^{k+1}\right) \leq \ldots \leq F\left(x_{j_\Lambda}^{k+1}\right).$$

As mentioned before, the μ best individuals are then selected for the new weighted mean m^{k+1}, which will shift the center of the particles' distribution towards the locations of the most favorable solutions:

$$\begin{aligned} m^{k+1} &= \sum_{i=1}^{\mu} w_i\, x_{j_i}^{k+1} = \sum_{i=1}^{\mu} w_i \left(m^k + \Omega^k B^k D^k z_{j_i}^{k+1}\right) \\ &= \underbrace{\sum_{i=1}^{\mu} w_i}_{=1} m^k + \sum_{i=1}^{\mu} w_i\, \Omega^k B^k D^k z_{j_i}^{k+1} = m^k + \Omega^k B^k D^k \sum_{i=1}^{\mu} w_i\, z_{j_i}^{k+1}. \end{aligned}$$

Subsequently, the evolution paths p_Ω^k, p_c^k and the step size Ω^k are updated:

$$p_\Omega^{k+1} = (1 - c_\Omega) p_\Omega^k + \sqrt{c_\Omega (2 - c_\Omega) \mu_{\text{eff}}} B^k (D^k)^{-1} (B^k)^T \left(\frac{m^{k+1} - m^k}{\Omega^k}\right),$$

$$p_c^{k+1} = (1 - c_c) p_c^k + h_\Omega^{k+1} \sqrt{c_c (2 - c_c) \mu_{\text{eff}}} B^k (D^k)^{-1} (B^k)^T \left(\frac{m^{k+1} - m^k}{\Omega^k}\right) \quad \text{and}$$

$$\Omega^{k+1} = \Omega^k \exp\left(\frac{c_\Omega}{d_\Omega}\left(\frac{\left\|p_\Omega^{k+1}\right\|_2}{\boldsymbol{E}[\|\mathcal{N}(0, \boldsymbol{I})\|_2]} - 1\right)\right), \quad \text{where}$$

$$h_\Omega^{k+1} = \begin{cases} 1, \text{ if } \dfrac{\left\|p_\Omega^{k+1}\right\|}{\sqrt{1 - (1 - c_\Omega)^{2(k+1)}}} < \left(\dfrac{3}{2} + \dfrac{1}{\text{Dim} - \frac{1}{2}}\right) \boldsymbol{E}[\|\mathcal{N}(0, \boldsymbol{I})\|_2] \\ 0, \text{ otherwise.} \end{cases}$$

The expected value belonging to a multivariate normal distribution with zero mean and covariance matrix $\boldsymbol{I}$ is typically approximated via

$$\boldsymbol{E}[\|\mathcal{N}(0, \boldsymbol{I})\|_2] \approx \sqrt{\text{Dim}}\left(1 - \frac{1}{4\text{Dim}} + \frac{1}{21\text{Dim}^2}\right).$$

Finally, the covariance matrix C^k is updated according to

$$\begin{aligned} C^{k+1} = {} & (1 - c_{\text{cov}}) C^k + \frac{c_{\text{cov}}}{\mu_{\text{cov}}}\left(p_c^{k+1} (p_c^{k+1})^T + \delta\left(h_\Omega^{k+1}\right) C^k\right) \\ & + c_{\text{cov}}\left(1 - \frac{1}{\mu_{\text{cov}}}\right) \sum_{i=1}^{\mu} w_i \left(\frac{x_{j_i}^{k+1} - m^k}{\Omega^k}\right)\left(\frac{x_{j_i}^{k+1} - m^k}{\Omega^k}\right)^T, \text{ where} \\ & \delta\left(h_\Omega^{k+1}\right) = \left(1 - h_\Omega^{k+1}\right) c_c (2 - c_c). \end{aligned}$$

The new matrix C^{k+1} determines the (co)variance of the particles' distribution in the next generation, while incorporating the knowledge about the location of the best function values from the current iteration. Together with the adjustments of m^k, this procedure aims at producing a population of particles that accumulate around the global optimum of F.

Our implementation included both the 2006 variant[18] of the algorithm as well as the newer version[17]. Prior to the application to the entire subject cohort mentioned in the main text, we compared both alternatives' performance on a set of a few random test subjects. Apart from the

population size Λ, we set all hyperparameters to the recommended default values and analyzed the results with respect to convergence speed, most favorable objective function values and the final spread of particles within a population (using criteria analogous to those considered for the cost function Ψ_{cost} in the main text). For Λ, we tested 8 different values between 6 (in the 2Dim parameter space) or 8 (in the 3Dim parameter space) and 144. It turned out that the 2006 version of the algorithm in combination with $\Lambda = 24$ particles was the most favorable and numerically stable option for the problems we consider.
We handled unfeasible solutions according to an algorithm-related boundary handling procedure[19]. The only alteration we made was that we intensified the boundary weights γ_i described in the article and worked with $\tilde{\gamma}_i = 5\,\gamma_i$.
The optimization procedure was stopped if the best objective function value discovered so far had not improved for 50 iterations or if a maximum of 80 iterations had been reached. This selection was made in analogy with the criteria chosen for Particle Swarm Optimization. Alternatives are described by Hansen[17]; a stricter termination criterion would be to stop already after $10 + \lfloor 3\ \text{Dim} / \Lambda \rfloor$ iterations without improvement, for example.

Bayesian Optimization

The minimization of the objective function F starts with the evaluation of a random sample of Λ points $x_1, \dots, x_\Lambda$ which may be uniformly distributed in X. A possible choice is $\Lambda \approx 10$ Dim as initial sample size[20,21], but lower values for Λ are not uncommon. The C++ software package *BayesOpt*[22] uses $\Lambda = 10$ and the MATLAB routine *bayesopt* (https://de.mathworks.com/help/stats/bayesopt.html) works with $\Lambda = 4$ as dimension-independent default value. To distinguish between the (constant) size of the initial sample and the (increasing) number of objective function evaluations computed so far, we denote the latter by $\tilde{\Lambda}$. Before the computation of new sampling points, both quantities are equal.
As a next step, a mean $m^{(0)}: X \to \mathbb{R}$ and kernel function have $K^{(0)}: X \times \mathrm{X} \to \mathbb{R}$ to be chosen. This is essential for Bayesian Optimization since the algorithm tries to approximate the function F, which may not be available in closed form, via a stochastic model. More concretely, a nonlinear process is used to model F, typically a Gaussian Process (GP)[23]. It can be interpreted as a quantity analogous to the function $F: X \to \mathbb{R}$, but instead of returning a scalar $F(x)$ for a certain $x \in X$, a GP returns the mean and (co)variance of a normal distribution over possible values of F at the point x. Formally, this dependence can be expressed as

$$m^{(0)}(x) = \boldsymbol{E}[F(x)] \ \text{ and } \ K^{(0)}(x, x') = \boldsymbol{E}\left[\left(F(x) - m^{(0)}(x)\right)\left(F(x') - m^{(0)}(x')\right)\right]$$

$$\Rightarrow F(x) \sim \mathcal{N}\left(m^{(0)}(x), \Theta^{(0)}(x)\right), \text{ where } \Theta^{(0)}(x) = K^{(0)}(x, x).$$

At this stage, the GP serves as a prior belief about F and its possible values.
It is common to choose a constant or relatively simple function for the mean[24], e.g. $m^{(0)}(x) = 0$. The kernel should be positive semi-definite and it should guarantee that two points x and x' lying close to each other in X are strongly correlated and thus generate a higher value $K^{(0)}(x, x')$ than two points that are further apart. One possible kernel is the *automatic relevance determination (ARD) squared exponential kernel* which is given by

$$K_{\text{SE}}(x, x') = \alpha_0 \exp\left(-\frac{1}{2}\right) r^2(x, x'), \text{where } r^2(x, x') = \sum_{i=1}^{\text{Dim}} \frac{x^i - x^{i\prime}}{\alpha_i}.$$

Note that we had to adapt our notation for this algorithm. Here, x^i, $i \in \{1, \dots, \text{Dim}\}$, denotes the i-th component of a point $x \in X$ (in the parameter optimization considered in the main text,

x^1 would be the coupling strength C, for example). In addition, the α_j, $j \in \{0, \ldots, \mathrm{Dim}\}$, represent the hyperparameters of the kernel, whose estimation we will discuss later below. The ARD squared exponential kernel has the advantage of being an uncomplicated function with the desired attributes. In practice, however, its usage in a GP generates approximations of the goal function F which are unrealistically smooth[25]. A useful alternative is therefore the *ARD Matérn 5/2 kernel* which is defined as

$$K_{\mathrm{M52}}(x, x') = \alpha_0 \left(1 + \sqrt{5r^2(x, x')} + \frac{5}{3} r^2(x, x') \right) \exp\left(-\sqrt{5r^2(x, x')} \right).$$

Other options for the kernel function can be found in algorithm-related works[24,26].

After having chosen the mean and kernel functions, the hyperparameters are optimized based on the knowledge that was obtained from the evaluation of the Λ initial sample points $x_1, \ldots, x_\Lambda$. Importantly, the procedures of this algorithm allow for more hyperparameters than $\alpha_0, \ldots, \alpha_{\mathrm{Dim}}$. On the one hand, it is possible to consider a constant mean $m^{(0)}(x) = \omega$ with unknown $\omega \in \mathbb{R}$, on the other hand, it can be accounted for the unknown observation noise ν in stochastic objective functions[24,25].

Summarizing all considered hyperparameters in a vector

$$\xi = (\alpha_0, \ldots, \alpha_{\mathrm{Dim}}, \omega, \nu),$$

the easiest approach is then to maximize the likelihood of the observations $x_1, \ldots, x_\Lambda$ (henceforth denoted by $x_{1:\Lambda}$) under the GP prior, i.e. for a given probability measure $\boldsymbol{P}$, we search for

$$\tilde{\xi} = \underset{\xi}{\mathrm{argmax}} \{ \boldsymbol{P}(F(x_{1:\Lambda}) | \xi) \},$$

which is known as the *maximum likelihood estimate* (MLE). Alternatively, it can be assumed that also ξ was chosen from a prior. The desired quantity $\tilde{\xi}$ is then given by the *maximum a posteriori estimate* (MAP)

$$\tilde{\xi} = \underset{\xi}{\mathrm{argmax}} \{ \boldsymbol{P}(\xi) | F(x_{1:\Lambda}) \} = \underset{\xi}{\mathrm{argmax}} \{ \boldsymbol{P}(F(x_{1:\Lambda}) | \xi) \boldsymbol{P}(\xi) \}.$$

It maximizes the posterior. The most exhaustive option is called the *fully Bayesian approach,* where an integration over all possible values of the hyperparameters is performed[24]. Further details on how to obtain the desired hyperparameters practically are provided in related papers[20,27].

The intention of all these considerations is to obtain an adequate prior distribution over possible functions for F. Based on the function evaluations obtained so far, Bayesian Optimization tries to generate the best possible surrogate model for F.

For a new sampling point $x_{\Lambda+1}$, the prior distribution of F is therefore given by

$$F(x_{\Lambda+1}) \sim \mathcal{N}\left(m^{(0)}(x_{\Lambda+1}), \Theta^{(0)}(x_{\Lambda+1}) \right)$$

along with the optimized hyperparameters of the (mean and) kernel. As described by Rasmussen and Williams[23], Bayes' theorem can then be applied in order to derive the posterior distribution of F given the previous observations:

$$F(x_{\Lambda+1}) \mid F(x_{1:\Lambda}) \sim \mathcal{N}\left(m^{(\Lambda)}(x_{\Lambda+1}), \Theta^{(\Lambda)}(x_{\Lambda+1}) \right).$$

The corresponding update formulas for the mean and kernel (variance) are

$$m^{(\Lambda)}(x) = K^{(0)}(x, x_{1:\Lambda}) \left(K^{(0)}(x_{1:\Lambda}, x_{1:\Lambda}) \right)^{-1} \left(F(x_{1:\Lambda}) - m^{(0)}(x_{1:\Lambda}) \right) + m^{(0)}(x),$$

$$\Theta^{(\Lambda)}(x) = K^{(0)}(x,x') - K^{(0)}(x,x_{1:\Lambda})\left(K^{(0)}(x_{1:\Lambda},x_{1:\Lambda})\right)^{-1} K^{(0)}(x_{1:\Lambda},x)$$

with $K^{(0)}(x,x_{1:\Lambda}) \in \mathbb{R}^{1\times\Lambda}$, $K^{(0)}(x_{1:\Lambda},x_{1:\Lambda}) \in \mathbb{R}^{\Lambda\times\Lambda}$, $F(x_{1:\Lambda}) \in \mathbb{R}^{\Lambda\times 1}$, $m^{(0)}(x_{1:\Lambda}) \in \mathbb{R}^{\Lambda\times 1}$ and $K^{(0)}(x_{1:\Lambda},x) \in \mathbb{R}^{\Lambda\times 1}$.

Having obtained the posterior distribution for possible values of $F(x_{\Lambda+1})$, the question arises what the most advantageous sampling point $x_{\Lambda+1}$ might be. In order to find it, Bayesian Optimization works with an acquisition function $a\colon X \to \mathbb{R}$ which provides an estimation about the area where the highest gain in terms of improved objective function values can be achieved. The acquisition function is typically designed in a way that aims at making it relatively easy to maximize. Since its evaluations are much cheaper to compute than those of the objective function F, standard techniques[27] can lead to a fast maximization of a. Frazier[24] recommended the quasi-Newton method L-BFGS-B[28], while the Nelder-Mead Algorithm[3] and other methods were tested by Schonlau and collaborators[21].
One popular approach is to consider the *expected improvement* as acquisition function. To compute it, we define $\tilde{F}$

$$\tilde{F} = \min\{F(x_1),\dots,F(x_\Lambda)\} \quad \text{and} \quad u(x) = \max\{0, \tilde{F} - F(x)\}$$

as the best value detected so far and the absolute improvement compared to it, respectively. Then, the acquisition function is given as

$$a_{\mathrm{EI}}(x) = \boldsymbol{E}[u(x)],$$

for which a closed form is available[20]. Another commonly used alternative is the *lower confidence bound* function

$$a_{\mathrm{LCB}}(x) = m^{(\Lambda)}(x) - \beta\sqrt{\Theta^{(\Lambda)}(x)}.$$

It creates a lower envelope of uncertainty around the posterior mean function whose width is given by the posterior standard deviation multiplied by a factor $\beta > 0$. For a minimization of F, -a_{LCB} is usually maximized. The parameter β can be adjusted to tune the balance between the desire for exploitation (sampling near favorable mean values) and exploration (sampling in areas of high uncertainty). MATLAB's routine *bayesopt* works with $\beta = 2$ as default value. Other possible acquisition functions are presented by Frazier[24].
When a new sampling point has been found, the corresponding objective function value is computed and then added to the list of previously obtained function evaluations. Afterwards, the hyperparameters of the initial mean and kernel function can be optimized again (with $\tilde{\Lambda}$ instead of Λ) or the posterior distribution over possible values of F is updated immediately based on the knowledge gained from the new sampling point. In the formulas, Λ will be replaced by $\tilde{\Lambda}$ then. Subsequently, the acquisition function is maximized again (this time with $\tilde{\Lambda}$) to find another sampling point. This procedure is repeated until a stopping criterion is met.
Since the matrix $K^{(0)}(x_{1:\Lambda},x_{1:\Lambda})$(or rather $K^{(0)}(x_{1:\tilde{\Lambda}},x_{1:\tilde{\Lambda}})$), whose size increases with every new sampling point, has to be inverted in each update step, it is recommended to either limit the number of maximum iterations to a moderate number or to gradually remove some of the initial points when a satisfactory amount of data has been collected[29]. From the viewpoint of numerical stability and performance, it may also be favorable to work with a Cholesky decomposition of the matrix[22].

For our implementation, we used the C++ software package *BayesOpt*[22] with $\Lambda = 5$ in the 2Dim parameter space and $\Lambda = 10$ in the 3Dim case. We chose a maximum of 80 iterations and worked with the default settings to handle unfeasible points. In addition, we also used the default to model the objective function F via a GP that features a constant mean function and the ARD

Matérn 5/2 kernel. Testing 8 combinations with different variants of hyperparameter learning methods and possible acquisition functions, we obtained favorable results for the fully Bayesian approach. It was used to optimize the kernel hyperparameters after every new function evaluation. Moreover, the idea of the lower confidence bound turned out as the most suitable approach for the acquisition function. All other settings were left at the default.

Supplementary References

1 Hicken, J., Juan, A. & Farhat, C. AA222 - Introduction to Multidisciplinary Optimization, Chapter 6: Gradient-Free Optimization. (2012). <http://adl.stanford.edu/aa222/Lecture_Notes.html>.

2 Lagarias, J. C., Reeds, J. A., Wright, M. H. & Wright, P. E. Convergence Properties of the Nelder--Mead Simplex Method in Low Dimensions. *SIAM Journal on Optimization* **9**, 112-147, doi:10.1137/s1052623496303470 (1998).

3 Nelder, J. A. & Mead, R. A Simplex Method for Function Minimization. *The Computer Journal* **7**, 308-313, doi:10.1093/comjnl/7.4.308 (1965).

4 Spendley, W., Hext, G. R. & Himsworth, F. R. Sequential Application of Simplex Designs in Optimisation and Evolutionary Operation. *Technometrics* **4**, 441-461, doi:10.1080/00401706.1962.10490033 (1962).

5 dfoptim: Derivative-Free Optimization v. R package version 2016.7-1 (2016).

6 Wessing, S. Proper initialization is crucial for the Nelder-Mead simplex search. *Optimization Letters* **13**, 847-856, doi:10.1007/s11590-018-1284-4 (2018).

7 Le Floc'h, F. Issues of Nelder-Mead Simplex Optimisation with Constraints. *SSRN Electronic Journal*, doi:10.2139/ssrn.2097904 (2012).

8 Box, M. J. A New Method of Constrained Optimization and a Comparison With Other Methods. *The Computer Journal* **8**, 42-52, doi:10.1093/comjnl/8.1.42 (1965).

9 Eberhart, R. & Kennedy, J. A new optimizer using particle swarm theory. *MHS'95. Proceedings of the Sixth International Symposium on Micro Machine and Human Science*, 39-43, doi:10.1109/MHS.1995.494215 (1995).

10 Kennedy, J. & Eberhart, R. Particle swarm optimization. *Proceedings of ICNN'95 - International Conference on Neural Networks* **4**, 1942-1948 vol.1944, doi:10.1109/ICNN.1995.488968 (1995).

11 Helwig, S. *Particle Swarms for Constrained Optimization / Partikelschwaerme fuer Optimierungsprobleme mit Nebenbedingungen, urn:nbn:de:bvb:29-opus-19334 (2010)*.

12 Perez, R. & Behdinan, K. Particle Swarm Optimization in Structural Design. *Swarm Intelligence, Focus on Ant and Particle Swarm Optimization*, doi:10.5772/5114 (2007).

13 Shi, Y. & Eberhart, R. A modified particle swarm optimizer. *1998 IEEE International Conference on Evolutionary Computation Proceedings. IEEE World Congress on Computational Intelligence (Cat. No.98TH8360)*, 69-73, doi:10.1109/ICEC.1998.699146 (1998).

14 Poli, R., Kennedy, J. & Blackwell, T. Particle swarm optimization. *Swarm Intelligence* **1**, 33-57, doi:10.1007/s11721-007-0002-0 (2007).

15 Banks, A., Vincent, J. & Anyakoha, C. A review of particle swarm optimization. Part I: background and development. *Natural Computing* **6**, 467-484, doi:10.1007/s11047-007-9049-5 (2007).

16 Eberhart, R. & Shi, Y. Particle swarm optimization: developments, applications and resources. *Proceedings of the 2001 Congress on Evolutionary Computation (IEEE Cat. No.01TH8546)* **1**, 81-86 vol. 81, doi:10.1109/CEC.2001.934374 (2001).

17 Hansen, N. The CMA Evolution Strategy: A Tutorial. *CoRR* **abs/1604.00772** (2016).
18 Hansen, N. in *Towards a New Evolutionary Computation: Advances in the Estimation of Distribution Algorithms* (eds Jose A. Lozano, Pedro Larrañaga, Iñaki Inza, & Endika Bengoetxea) 75-102 (Springer Berlin Heidelberg, 2006).
19 Hansen, N., Niederberger, A. S. P., Guzzella, L. & Koumoutsakos, P. A Method for Handling Uncertainty in Evolutionary Optimization With an Application to Feedback Control of Combustion. *IEEE Transactions on Evolutionary Computation* **13**, 180-197, doi:10.1109/tevc.2008.924423 (2009).
20 Jones, D. R., Schonlau, M. & Welch, W. J. Efficient Global Optimization of Expensive Black-Box Functions. *Journal of Global Optimization* **13**, 455-492, doi:10.1023/a:1008306431147 (1998).
21 Schonlau, M., Welch, W. J. & Jones, D. R. Global versus local search in constrained optimization of computer models. *Institute of Mathematical Statistics Lecture Notes - Monograph Series* **34**, 11-25, doi:10.1214/lnms/1215456182 (1998).
22 Martinez-Cantin, R. BayesOpt: A Bayesian Optimization Library for Nonlinear Optimization, Experimental Design and Bandits. *Journal of Machine Learning Research* **15**, 3735-3739 (2014).
23 Rasmussen, C. E. & Williams, C. K. I. *Gaussian Processes for Machine Learning*. (The MIT Press, 2006).
24 Frazier, P. I. Bayesian Optimization. *Recent Advances in Optimization and Modeling of Contemporary Problems*, 255-278, doi:10.1287/educ.2018.0188 (2018).
25 Snoek, J., Larochelle, H. & Adams, R. P. Practical Bayesian optimization of machine learning algorithms. *Proceedings of the 25th International Conference on Neural Information Processing Systems* **2**, 2951-2959 (2012).
26 Brochu, E., Cora, V. M. & de Freitas, N. A Tutorial on Bayesian Optimization of Expensive Cost Functions, with Application to Active User Modeling and Hierarchical Reinforcement Learning. *ArXiv* **abs/1012.2599** (2010).
27 Bull, A. D. Convergence Rates of Efficient Global Optimization Algorithms. *J. Mach. Learn. Res.* **12**, 2879-2904 (2011).
28 Liu, D. C. & Nocedal, J. On the limited memory BFGS method for large scale optimization. *Mathematical Programming* **45**, 503-528, doi:10.1007/bf01589116 (1989).
29 Rasmussen, C. E. Gaussian Processes to Speed up Hybrid Monte Carlo for Expensive Bayesian Integrals. *Bayesian Statistics 7*, 651-659 (2003).
